# Distinct Longitudinal Brain White Matter Microstructure Changes and Associated Polygenic Risk of Common Psychiatric Disorders and Alzheimer’s Disease in the UK Biobank

**DOI:** 10.1016/j.bpsgos.2024.100323

**Published:** 2024-04-26

**Authors:** Max Korbmacher, Dennis van der Meer, Dani Beck, Daniel E. Askeland-Gjerde, Eli Eikefjord, Arvid Lundervold, Ole A. Andreassen, Lars T. Westlye, Ivan I. Maximov

**Affiliations:** a Department of Health and Functioning, Western Norway University of Applied Sciences, Bergen, Norway; b NORMENT Centre for Psychosis Research, Division of Mental Health and Addiction, University of Oslo and Oslo University Hospital, Oslo, Norway; c Mohn Medical Imaging and Visualization Centre, Bergen, Norway; d Faculty of Health, Medicine and Life Sciences, Maastricht University, Maastricht, The Netherlands; e Department of Psychiatric Research, Diakonhjemmet Hospital, Oslo, Norway; f Department of Psychology, University of Oslo, Oslo, Norway; g Department of Biomedicine, University of Bergen, Bergen, Norway; h KG Jebsen Centre for Neurodevelopmental Disorders, University of Oslo, Oslo, Norway

**Keywords:** Aging, Diffusion MRI, Magnetic resonance imaging, Microstructure, Polygenic risk, White matter

## Abstract

**Background:**

During the course of adulthood and aging, white matter (WM) structure and organization are characterized by slow degradation processes such as demyelination and shrinkage. An acceleration of such aging processes has been linked to the development of a range of diseases. Thus, an accurate description of healthy brain maturation, particularly in terms of WM features, is fundamental to the understanding of aging.

**Methods:**

We used longitudinal diffusion magnetic resonance imaging to provide an overview of WM changes at different spatial and temporal scales in the UK Biobank (UKB) (*n* = 2678; age_scan 1_ = 62.38 ± 7.23 years; age_scan 2_ = 64.81 ± 7.1 years). To examine the genetic overlap between WM structure and common clinical conditions, we tested the associations between WM structure and polygenic risk scores for the most common neurodegenerative disorder, Alzheimer’s disease, and common psychiatric disorders (unipolar and bipolar depression, anxiety, obsessive-compulsive disorder, autism, schizophrenia, attention-deficit/hyperactivity disorder) in longitudinal (*n* = 2329) and cross-sectional (*n* = 31,056) UKB validation data.

**Results:**

Our findings indicate spatially distributed WM changes across the brain, as well as distributed associations of polygenic risk scores with WM. Importantly, brain longitudinal changes reflected genetic risk for disorder development better than the utilized cross-sectional measures, with regional differences giving more specific insights into gene-brain change associations than global averages.

**Conclusions:**

We extend recent findings by providing a detailed overview of WM microstructure degeneration on different spatial levels, helping to understand fundamental brain aging processes. Further longitudinal research is warranted to examine aging-related gene-brain associations.

## Plain Language Summary

In their study, Korbmacher *et al.* benchmark healthy aging processes in the brain’s white matter. Findings of degrading white matter at higher ages were consistent with recent cross-sectional and longitudinal findings, particularly outlining changes in ventricle-near and cerebellar white matter. Degenerative processes were also found to accelerate at a higher age. Finally, the polygenic risk to develop psychiatric and neurodegenerative disorders was weakly associated with the white matter change in the otherwise healthily aging participants.

White matter microstructure (WMM) changes significantly throughout the life span (1, 2, 3, 4, 5, 6, 7). Recent large-scale studies suggest a strong association between WMM and age in both healthy and diseased aging. Previous findings demonstrated general trends of tissue anisotropy increases and water diffusivity decreases throughout childhood (8, 9, 10), and, as reversal dynamics of these trends, throughout adulthood (3,11). Importantly, abnormal WM development has been associated with the development of neurocognitive skills and mental health symptoms in childhood and adolescence (12) and brain disorders later in life (13,14). A person’s genetically determined propensity to develop a certain disorder can also be summarized by polygenic risk scores (PRSs) (15, 16, 17, 18, 19, 20, 21, 22, 23). Combining brain imaging and genetics provides an opportunity to associate the elevated genetic risk for disorders with specific brain features in terms of scalar imaging metrics (24,25). This allows identification of which brain regions may be more prone to disease development based on genetic makeup and provides additional biological detail to the observed WM changes. An advantage of using WMM for such associations is the level of detail provided by biophysical models, such as intra- and extra-axonal diffusion processes (26, 27, 28, 29, 30). WM-specific changes are associated with various common psychiatric disorders (13,14), and these changes precede, for example, symptom onset in Alzheimer’s disease (AD) (31). This identifies WMM as an important aspect for further investigation in disease formation and outcomes.

Because the temporal aspect matters when investigating tissue changes, longitudinal designs are required (6). Therefore, longitudinal changes in WM are informative when examining PRS-tissue associations because they allow us to connect PRSs with actual WM change and not only cross-sectional snapshots of brain structure at a given moment of an individual’s life.

To estimate aging effects on WM alterations, studies commonly focus on diffusion tensor imaging (DTI) (32). While DTI is useful for mesostructural characterizations, it is also limited in addressing certain common phenomena in WM such as crossing fiber bundles, non-Gaussian diffusion, and differences between intra- and extra-axonal water compartments (33). Recent achievements in advanced diffusion magnetic resonance imaging (dMRI) techniques offer a spectrum of biophysical models (26, 27, 28, 29, 30) addressing these issues, for example, by differentiating between intra- and extra-axonal space (26,28,30) or by capturing non-Gaussian diffusion (26,27). In turn, there are few longitudinal studies in which WMM changes have been observed (10,34,35) and even fewer that also utilized advanced diffusion approaches beyond DTI (6,10).

To fill this gap, we assessed the metrics of a series of dMRI approaches in a large longitudinal mid-to-late life adult sample provided by the UK Biobank (UKB) (36) and identified the spatiotemporal patterns of aging-related WMM changes on a global, regional, and voxel-level scale. To further investigate potential genetic underpinnings of these WMM changes, we estimated PRSs informed by previous genome-wide association studies for AD, the most common neurodegenerative disorder, and common psychiatric disorders, including major depressive disorder (MDD), bipolar disorder (BIP), anxiety disorder (ANX), autism spectrum disorder (ASD), schizophrenia (SCZ), attention-deficit/hyperactivity disorder (ADHD), and obsessive-compulsive disorder (OCD). PRSs capture an individual’s genetic propensity for a trait by aggregating the estimated effects of risk variants across the entire genome. Together with brain structure and brain structural changes, PRSs may be informative for the development of disease. For example, these gene-brain associations can help identify concrete spatial patterns for different diseases (37,38). For further inference on the generalizability of the associations between PRSs and WM in the longitudinal sample with available PRSs (after exclusions: *n* = 2329), we estimated the same associations for independent participants from the cross-sectional portion of the UKB (after exclusions: *n* = 31,056) (36). Based on previous findings (3,39), we expected near-linear age associations of diffusion metrics, generally outlining lower fractional anisotropy, intra-axonal water fraction, and kurtosis at higher ages, but higher diffusivity and extra-axonal free water fractions. Moreover, we expected to observe PRS-WMM associations for AD globally and in most age-sensitive regions.

## Methods and Materials

### Sample Characteristics

We obtained UKB data (36), including the longitudinal dMRI data of 4871 participants at 2 time points. Participant data were excluded when consent had been withdrawn and when dMRI data did not meet quality control standards using the YTTRIUM method (40) (also see Supplemental Appendix A). Additionally, we excluded participants who were diagnosed with any mental and behavioral disorder (ICD-10 category F), disease of the nervous system (ICD-10 category G), or disease of the circulatory system (ICD-10 category I). After exclusion criteria were applied, there were 2678 participants (52.99% female). At baseline, the mean age of participants was 62.26 ± 7.19 years (range: 46.12–80.30 years), and at time point 2, the mean age was 64.70 ± 7.07 years (range: 49.33–82.59 years), indicating an average age difference as Δ*age* = 2.44 ± 0.73 years (range: 1.12–6.90 years). The data were collected at 3 sites: 1) Cheadle (57.36%), 2) Newcastle (37.04%), and 3) Reading (5.60%). PRS data were available for 2329 of these longitudinal datasets and for 31,056 cross-sectional validation datasets (after exclusions). We highlight the fact that the UKB is a relatively homogeneous sample, containing mainly White UK citizens.

### MRI Acquisition and Postprocessing

UKB MRI data acquisition procedures are described elsewhere (36,41,42).

After obtaining access to the raw dMRI data, we preprocessed it using an optimized pipeline. The pipeline includes corrections for noise (43), Gibbs ringing (44), susceptibility-induced and motion distortions (45), and eddy currents artifacts. Isotropic 1 mm^3^ Gaussian smoothing was carried out using FSL’s *fslmaths*. Using the multi-shell data, DTI (32), diffusion kurtosis imaging (DKI) (27), and white matter tract integrity (26) metrics were estimated using MATLAB version 2017b (The MathWorks, Inc.) code (https://github.com/NYU-DiffusionMRI/DESIGNER). Spherical mean technique (SMT) (29) and multi-compartment spherical mean technique (28) metrics were estimated using original code (https://github.com/ekaden/smt). Estimates from the Bayesian rotational invariant approach (BRIA) (30) were evaluated by the original MATLAB code (https://bitbucket.org/reisert/baydiff/src/master/).

In total, we obtained 26 WM metrics from 6 diffusion approaches (DTI, DKI, WM tract integrity, SMT, multi-compartment SMT, BRIA) (for an overview, see Supplemental Appendix B). To normalize all metrics, we used tract-based spatial statistics (46), as part of FSL (47,48). In brief, initially all brain-extracted (49) fractional anisotropy (FA) images were aligned to Montreal Neurological Institute space using nonlinear transformation (FNIRT). Thereafter, the mean FA image and related mean FA skeleton were derived. Each diffusion scalar map was projected onto the mean FA skeleton using tract-based spatial statistics. To provide a quantitative description of diffusion metrics at a region level, we used the Johns Hopkins University atlas (50) and obtained 30 hemisphere-specific WM regions of interest (ROIs) based on a probabilistic WM atlas (Johns Hopkins University) (51) for each of the 26 metrics. Altogether, 1794 diffusion features were derived per individual (26 metrics × [48 ROIs + 20 tracts + 1 global mean value]).

### Polygenic Risk Scores

We estimated PRSs for each participant with available genomic data, using PRSice2 with default settings. As input for the PRSs, we used summary statistics from recent genome-wide association studies of ASD, MDD, SCZ, ADHD, BIP, OCD, ANX, and AD. We used a minor allele frequency of 0.05 as the most commonly used threshold across PRS studies of psychiatric disorders.

While psychiatric disorders were *p-*value thresholded at α = 0.05 (15, 16, 17, 18, 19, 20, 21, 22, 23), recommendations for AD (α = 1.07^−4^) (52) led to the application of a lower threshold of α = 0.0001, with the goal of optimizing signal to noise in comparison to previously used α = 0.001 (53). The goal with the estimation of the PRSs was to relate cross-sectional WMM metrics and WMM changes to disease-related genetic profiles to examine to which degree these genetic risk profiles can explain WMM changes in midlife to senescence.

### Statistical Analyses

All statistical analyses were carried out using R version 4.2.0 (www.r-project.org) and FSL version 6.0.1 (48).

First, we assessed unadjusted time point differences by using paired samples *t* tests on a set of cognitive measures and each of the global and regional scalar diffusion metrics *F* (such as FA from DTI) and present Cohen’s *d* indicating the effect size (equation 1):

(1) $$d=\frac{{\bar{x}}_{1}-{\bar{x}}_{2}}{\sigma }$$

describing the difference between means (${\bar{x}}_{1}$, ${\bar{x}}_{2}$) over an estimate of the sample standard deviation of the data (σ).

Then we used linear mixed-effects regression models adjusting *F* for age, sex, the sex-by-age interaction (sex × age), time point (TP), and scanner site (site). ID was treated as random intercept (RI), and *y* the y intercept (equation 2):

(2) $$\hat{F}=y+\beta_{0}\times Age+\beta_{1}\times Sex+\beta_{2}\times Age\times Sex+\beta_{3}\times TP+\beta_{4}\times Site+RI\left(ID\right)$$

For comparison of age relationships between time points, we used simple linear and generalized additive models on each single time point (treating the data as cross-sectional), with the linear models taking the following form (equation 3):

(3) $$\hat{F}=y+\beta_{0}\times Age+\beta_{1}\times Sex+\beta_{2}\times Age*Sex+\beta_{3}\times Site\text{,}$$

and the generalized additive models taking this form (including a spline function of age, S(Age), to model nonlinear associations) (equation 4):

(4) $$\hat{F}=y+S\left(Age\right)+\beta_{1}\times Sex+\beta_{2}\times Age*Sex+\beta_{3}\times Site\text{.}$$

We also estimated the annual rate of change (ARoC) of each global feature by taking the difference in WMM features *F* between time points over the time passed between time points (scans 1 and 2) indicated by the Δ*age* = *age*_*scan*2_ − *age*_*scan*1_ (equation 5):

(5) $$\begin{array}{r} ARoC=\frac{F_{scan2}-F_{scan1}}{\Delta age}\text{.} \end{array}$$

Global ARoC was corrected for sex, sex × age, and scanner site (as RI) (equation 6):

(6) $${\hat{ARoC}}_{global}=y+\beta_{0}\times Age*Sex+\beta_{1}\times Sex+RI\left(Site\right)\text{,}$$

for which then age correlations were estimated. For regional features, we used simple linear models to support model convergence (equation 7):

(7) $${\hat{ARoC}}_{regional}=y+\beta_{0}\times Age*Sex+\beta_{1}\times Sex+\beta_{2}\times Age+\beta_{3}\times Site\text{.}$$

For the voxel-level analysis, we estimated one-sample *t* tests on the contrast between each time point’s maps within the FA skeleton accounting for age, Δage, scanner site, and sex using FSL randomise with 10,000 permutations (H_0_: difference = 0).

Finally, we assessed the associations between ARoC and PRS for global and regional WMM metrics adjusting for age, age × sex, and site using simple linear models (equation 8):

(8) $$\hat{ARoC}=y+\beta_{0}\times Age*Sex+\beta_{1}\times Age+\beta_{2}\times Sex+\beta_{3}\times Site+\beta_{4}\times PRS\text{.}$$

For voxel-level analyses, we used tract-based spatial statistics randomise with permutation-based statistics (running 10,000 permutations) (54). Mean maps and between-time-point contrast maps served for the computation of one-sample *t* tests for each of the observed metric, while accounting for age, sex, and site (i.e., random intercept models) (equation 9):

(9) $$\hat{F}=y+\beta_{0}\times Age+\beta_{1}\times Sex+\beta_{2}\times Site+\beta_{3}\times TP+RI\left(ID\right)\text{.}$$

*p* Values were adjusted for multiple comparison using Bonferroni correction (55) for global and region-averaged metrics, and familywise error corrections were used for voxelwise inferential statistics [using threshold-free cluster enhancement (56)]. We report conditional variance explained in the main text ($R_{c}^{2}$), which refers to variance explained by fixed factors.

## Results

### Cognitive Changes

Our analysis suggested no significant time point differences in cognitive measures for the interscan interval of $\bar{\Delta }$ = 2.44 ± 0.73 (Supplemental Appendix P).

### Global WMM Changes

The globally averaged WMM metrics differed between time points (Cohen’s $\left|\bar{d}\right|=0.073\;\pm \;0.055,d_{\min}=-\;0.248,d_{\max}=0.159$), with the exception of BRIA–micro apparent diffusion coefficient, all SMT metrics, and multi-compartment SMT–diffusion coefficient (Appendices C and D).

Congruently, linear mixed-effects regression model (equation 2) outlines the effect of time point, in addition to age and sex (Supplemental Appendix Ea,b). While the effects of time point ($\left|\bar{\beta }\right|=0.078\;\pm \;0.024,\beta_{\min}=-\;0.107,\beta_{\max}=0.113$), age ($\left|\bar{\beta }\right|=0.047\;\pm \;0.014,\;\beta_{\min}=-\;0.063,\beta_{\max}=0.067$), and sex × age ($\left|\bar{\beta }\right|=0.26\;\pm \;0.182,\beta_{\min}=-\;0.619,\beta_{\max}=0.197$) were significant (*p* < .001) (Appendices F and G), and modeling diffusion metrics well ${\bar{R^{2}}}_{c}=88.40\%\;\pm \;7.33\%$ (Supplemental Appendix E), only 2 diffusion metrics showed significant sensitivity to sex (BRIA micro FA: β = −0.642, *p* = .034, DKI radial kurtosis: β = −0.785, *p* = .022, both lower in males), and presented large variability (Supplemental Appendix Ec, d and Supplemental Appendices I and J).

**Figure 1:**
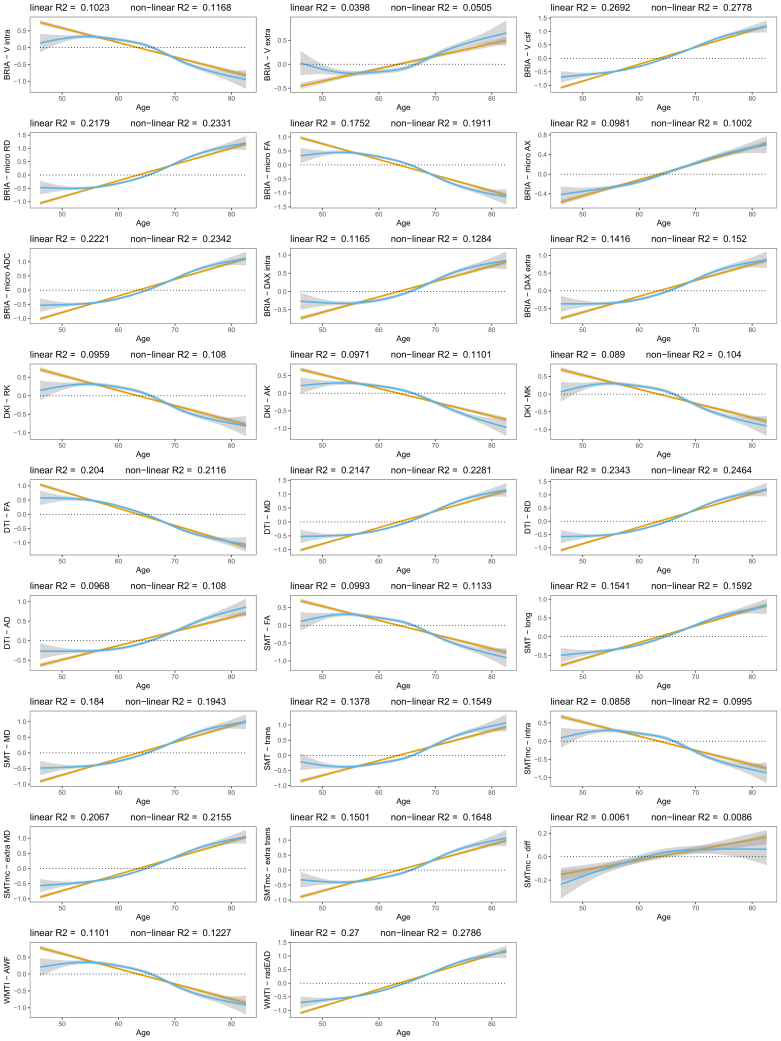
Global white matter microstructure (WMM) aging trajectories. First, WMM values were standardized, mean centered, and adjusted for covariates of no interest using linear mixed models. Second, age-WMM relationships were described by linear and nonlinear functions. An overview of the utilized diffusion approaches and entailed WMM metrics can be found in Supplemental Appendix B. AD, axial diffusivity; ADC, apparent diffusion coefficient; AK, axial kurtosis; AWF, axonal water fraction; BRIA, Bayesian rotational invariant approach; DAX extra, extra-axonal axial diffusivity; DAX intra, intra-axonal axial diffusivity; DKI, diffusion kurtosis imaging; DTI, diffusion tensor imaging; FA, fractional anisotropy; mcSMT, multi-compartment spherical mean technique; MD, mean diffusivity; micro AX, microscopic axial diffusivity; MK, mean kurtosis; radEAD, radial extra-axonal diffusivity; RD, radial diffusivity; RK, radial kurtosis; SMT, spherical mean technique; Vcsf, cerebrospinal fluid fraction; V extra, extra-axonal water fraction; V intra, intra-axonal water fraction; WMTI, white matter tract integrity.

Moreover, WMM metrics’ age associations were better modeled as nonlinear ($\bar{R^{2}}=15.81\%\;\pm \;6.88\%$) than linear associations ($\bar{R^{2}}=14.69\%\;\pm \;6.84\%$), considering variance explained (Figure 1). Yet, this difference in model fit was nonsignificant (*p* = .557). We showed decreases in FA and kurtosis ($\left|\bar{\beta }\right|=-\;0.043\;\pm \;0.009,\;\beta_{\min}=-\;0.063,\;\beta_{\max}=\;\;-\;0.035$). At the same time, water diffusivity and extra-axonal water fraction, including free water (mainly cerebrospinal fluid) increased ($\left|\bar{\beta }\right|=0.052\;\pm \;0.013,\beta_{\min}=0.025,\beta_{\max}=0.067$). Notably, the intra-axonal water fraction, estimated by different diffusion approaches, exhibited the same behavior independent of the diffusion approach (lower at higher ages).

**Figure 2:**
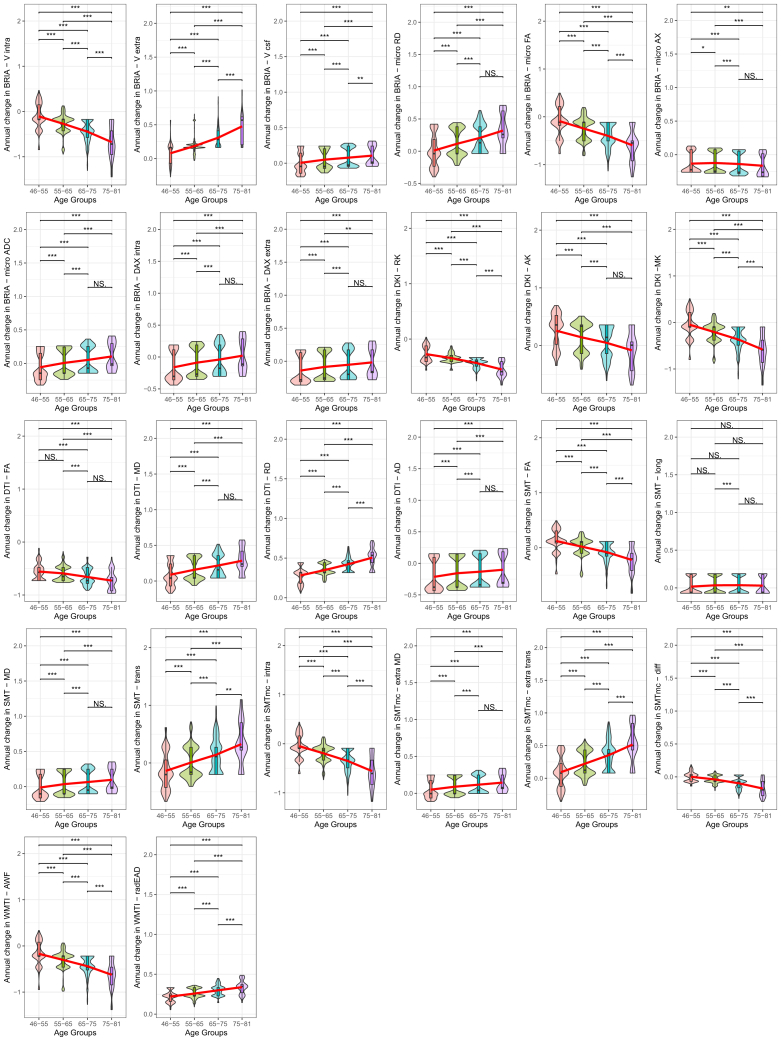
Age-stratified annual white matter microstructure change. White matter microstructure was corrected for age, sex, age × sex, and site and standardized for comparability (without mean centering). We present *p* values for Wilcoxon tests, which were significant at the Bonferroni-corrected ∗*α* < .05/(26 × 6) = 3.21 × 10^−^^4^, ∗∗*α* < 0.01/(26 × 6) = 6.41 × 10^−^^5^, ∗∗∗*α* < 0.001/(26 × 6) = 6.41 × 10^−^^6^. The red lines were added as a visual aid to identify trends of accelerated or decelerated annual change. An overview of the utilized diffusion approaches and entailed white matter microstructure metrics can be found in Supplemental Appendix B. AD, axial diffusivity; ADC, apparent diffusion coefficient; AK, axial kurtosis; AWF, axonal water fraction; BRIA, Bayesian rotational invariant approach; DAX extra, extra-axonal axial diffusivity; DAX intra, intra-axonal axial diffusivity; DKI, diffusion kurtosis imaging; DTI, diffusion tensor imaging; FA, fractional anisotropy; mcSMT, multi-compartment spherical mean technique; MD, mean diffusivity; micro AX, microscopic axial diffusivity; MK, mean kurtosis; NS, nonsignificant; radEAD, radial extra-axonal diffusivity; RD, radial diffusivity; RK, radial kurtosis; SMT, spherical mean technique; Vcsf, cerebrospinal fluid fraction; V extra, extra-axonal water fraction; V intra, intra-axonal water fraction; WMTI, white matter tract integrity.

Finally, we present accelerations of the ARoC at higher ages for most global WMM metrics (Figure 2). Large age-group differences were observed at Cohen’s $\left|{\bar{d}}_{sign}\right|=0.802\;\pm \;0.453,\left|d_{\min}\right|=0.015,\left|d_{\max}\right|=1.920$ (when including also nonsignificant findings: $\left|{\bar{d}}_{all}\right|=0.732\;\pm \;0.477,\left|d_{\min}\right|=0.010,\left|d_{\max}\right|=1.92$ (see Supplemental Appendix R for details on test statistics, including effect size estimates). Decelerating ARoC was observed for BRIA- intra and extra axonal axial diffusivity (DAX intra and extra), and DKI-axial kurtosis, and relatively stable ARoC for SMT–longitudinal coefficient. See also Supplemental Appendix S for ARoC-age trends indicating accelerated aging at ${\bar{\beta }}_{sig}=0.012$, Supplemental Appendix S for uncorrected age stratification, and Supplemental Appendix T for corrected sex stratification.

### Regional WMM Changes

**Figure 3:**
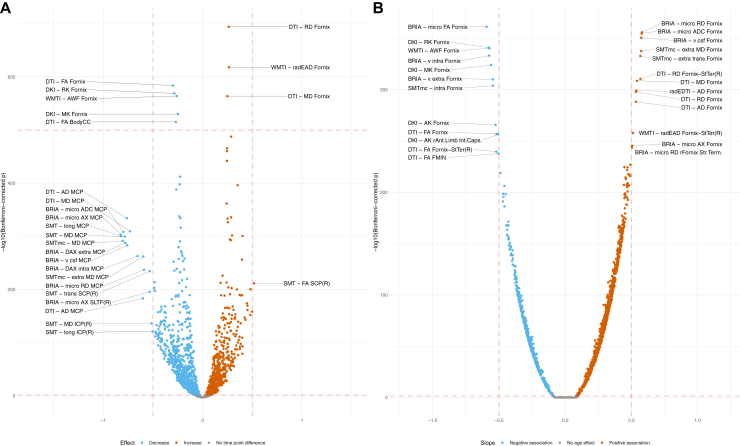
Regional white matter microstructure changes between time points and age associations. **(A)** Unadjusted effect sizes (Cohen’s *d*) vs. Bonferroni-adjusted −log_10_*p* values. Labeling was done using a medium effect size threshold of Cohen’s |*d*| > 0.5 (also marked with vertical lines) as well as extreme Bonferroni-adjusted *p* values of −log_10_(*p*) > 500. **(B)** Adjusted white matter microstructure associations with age. Age–white matter microstructure were adjusted for sex, sex × age, scanner site, and time point (equation 2). The plot presents standardized slopes (*β*) vs. Bonferroni-adjusted −log_10_*p* values. Labeling was done using a large association of |*β*| > 0.5 (also marked with vertical lines). Dotted lines were inserted as visual aid: the lower horizontal dotted line represent the significance level of α = .05 and the upper horizontal line −log_10_(*p*) = 500. The vertical lines represented labeling borders based on a medium effect size of Cohen’s |*d*| > 0.5 **(A)** and large associations of |*β*| > 0.5 **(B)**. Tables with test statistics are available at https://github.com/MaxKorbmacher/Long_Diffusion/. AD, axial diffusivity; AK, axial kurtosis; AWF, axonal water fraction; BodyCC, body of the corpus callosum; BRIA, Bayesian rotational invariant approach; DAX extra, extra-axonal axial diffusivity; DAX intra, intra-axonal axial diffusivity; DKI, diffusion kurtosis imaging; DTI, diffusion tensor imaging; FA, fractional anisotropy; FMIN, Forceps Minor; ICP, inferior cerebellar peduncle; MCP, middle cerebellar peduncle; mcSMT, multi-compartment spherical mean technique; MD, mean diffusivity; microAX, microscopic axial diffusivity; MK, mean kurtosis; radEAD, extra-axonal radial diffusivity; RD, radial diffusivity; RK, radial kurtosis; SCP(R), right superior cerebellar peduncle; SLTF(R), right superior longitudinal temporal fasciculus; SMT, spherical mean technique; StTer(R), right stria terminalis; Vcsf, cerebrospinal fluid fraction; V extra, extra-axonal water fraction; V intra, intra-axonal water fraction.

Estimating paired samples tests indicate that 47.57% of WM features decreased ($\left|{\bar{d}}_{sign}\right|=-0.191\;\pm \;0.122,\left|d_{\min}\;\right|=\;-\;0.825,\;\left|d_{\max}\right|=-\;0.030$), 32.30% increased ($\left|{\bar{d}}_{sign}\right|=0.159\;\pm \;0.091,\left|d_{\min}\right|=0.283,\left|d_{\max}\right|=0.516$) and 20.13% did not change between time points. Most extreme *p* values among these unadjusted time point differences were observed in the fornix (DTI-radial diffusivity: $d_{p_{\min}}=0.262;\;95\%\;\text{CI},0.252–0.271$) and the body of the corpus callosum (DTI-FA: $d_{p_{\min}}=-\;0.270;\;95\%\;\text{CI},-\;0.281\;\text{to}-\;0.259$), and largest effect sizes of Cohen’s |*d*| > 0.5 found in the middle cerebellar peduncle (BRIA microAX: *d*_*max*_ = −0.825; 95% CI, −0.875 to −0.775) (Figure 3A).

Linear mixed-effects regression model (equation 2) outlined strongest age associations in fornix WMM (|*d*| > 0.5) (Figure 3B) (see Supplemental Appendix J for distribution of β values). We also identified various regions sensitive to sex differences and smaller sex × age interaction effects, with repeated occurrence of significant fornix, cerebral peduncle, corticospinal tract, and medial lemniscus differences (Supplemental Appendices H and I). Across regions, time point was a nonsignificant fixed effect (β < 1.5 × 10^−5^, *p* > .05). For comparability across regions, we used |*ARoCs*| in association with age, adjusting for age × sex, sex, and site effects, showing accelerated aging (${\bar{\beta }}_{\text{sig}}=0.013\;\pm \;0.005$) (Supplemental Appendix K).

### Voxelwise WMM Changes

When investigating voxelwise changes of WMM between time points (adjusted for age, sex, and scanner site), we found 2 major patterns examining both increases and decreases in WMM: 1) a global decrease in fractional anisotropy metrics (with the exception of SMT-FA showing distributed changes, including frontal increase and posterior decrease), and 2) an overall decrease in radial, axial, and mean diffusivity metrics in orbitofrontal, occipital, and brain stem and cerebellum and increase in superior frontal areas (Table 1). For an overview of the voxelwise WMM maps at corrected α = 0.05, see Supplemental Appendix Y.

**Table 1:** Qualitative assessment of gradients of white matter change identified by voxel-based analyses

Model	Metric	Superior Frontal	Orbito-Frontal	Posterior	Cerebellum and Brain Stem
BRIA	Dax extra	↑	↑↓	↓	↓
	Dax intra	↑	↑↓	↓	↓
	DRAD extra^a^	↑	↑↓	↓	↓
	Micro ADC	↑	↑↓	↓	↓
	Micro AX	↑	↑↓	↓	↑↓
	Micro FA	↓	↑↓	↑↓	↑↓
	Micro RD	↑	↑↓	↓	↑↓
	Vcsf	↑	↑↓	↓	↑↓
	Vextra	↓	↑↓	↑	↑↓
	Vintra	↑↓	↑↓	↓	↑↓
DKI	AK	↓	↑	↑↓	↑↓
	MK	↑↓	↑↓	↓	↑↓
	RK	↑↓	↑↓	↓	↓
DTI	AD	↑	↓	↓	↑↓
	FA	↓	↓	↓	↑↓
	MD	↑	↑	↓	↓
	RD	↑	↑	↑↓	↓
SMT	FA	↑	↑↓	↓	↑↓
	MD	↑	↑↓	↓	↓
	Long	↑	↑↓	↓	↓
	Trans	↑↓	↑↓	↑↓	↑↓
mcSMT	Diffusion	↑	↑↓	↓	↑↓
	Extra MD	↑	↑↓	↓	↑↓
	Extra trans	↑	↑↓	↑↓	↑↓
	Intra	↑↓	↑↓	↓	↑↓
WMTI	AWF	↑↓	↑↓	↓	↑↓
	axEAD^a^	↑	↑↓	↓	↑↓
	radEAD	↑	↑↓	↑↓	↑↓

↑ indicates a general trend of increases, and ↓ of decreases. ↑↓ indicates trends of both increases and decreases.

AD, axial diffusivity; ADC, apparent diffusion coefficient; AK, axial kurtosis; AWF, axonal water fraction; axEAD, axial extra axonal diffusivity; BRIA, Bayesian rotational invariant approach; DAX extra, extra-axonal axial diffusivity; DAX intra, intra-axonal axial diffusivity; DKI, diffusion kurtosis imaging; Drad extra, extra axonal radial diffusivity; DTI, diffusion tensor imaging; FA, fractional anisotropy; mcSMT, multi-compartment spherical mean technique; MD, mean diffusivity; micro AX, microscopic axial diffusivity; MK, mean kurtosis; RD, radial diffusivity; RK, radial kurtosis; SMT, spherical mean technique; Vcsf, cerebrospinal fluid fraction; V extra, extra -axonal water fraction; V intra, intra-axonal water fraction; WMTI, white matter tract integrity.

a Note that Drad extra from the BRIA and the axial extra axEAD metrics from the WMTI approach were excluded from the analyses as a significant portion of the produced metrics did not pass our quality control procedure. An overview of the utilized diffusion approaches and entailed white matter microstructure metrics can be found in Supplemental Appendix B. Voxel maps can be found in Supplemental Appendix Y.

### PRS Associations

**Figure 4:**
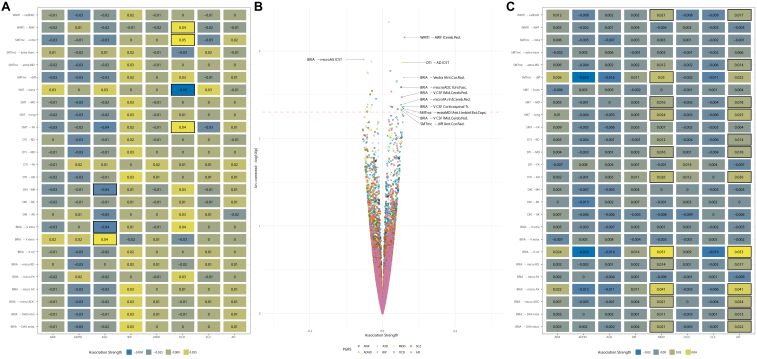
Associations of polygenic risk scores (PRSs) with the rate of white matter change. **(A)** The global associations between PRS and white matter microstructure change. Colors indicate the strength of association (standardized β coefficients). **(B)** Regional associations between PRS and white matter microstructure change. The dotted line indicates an uncorrected α < 0.001. Labels presenting the respective regions and metrics are supplied above this α threshold, as well as at |*β*| > 0.05. **(C)** Regional associations exclusively for the medial cerebral peduncle, the region where the strongest annual rate of change-PRS associations were observed. Boxes in **(A)** and **(C)** indicate the statistical significance at an uncorrected α < 0.05. All associations were adjusted for age, sex, the age × sex interaction, and site. None of the presented associations survived the adjustment of the α level for multiple comparisons. An overview of the utilized diffusion approaches and entailed white matter microstructure metrics can be found in Supplemental Appendix B. AD, Alzheimer’s disease; ADC, apparent diffusion coefficient; ADHD, attention-deficit/hyperactivity disorder; AK, axial kurtosis; ANX, anxiety; ASD, autism spectrum disorder; AWF, axonal water fraction; BIP, bipolar disorder; BRIA, Bayesian rotational invariant approach; DAX extra, extra-axonal axial diffusivity; DAX intra, intra-axonal axial diffusivity; DTI - AD, diffusion tensor imaging - axial diffusivity; FA, fractional anisotropy; lCST, left cerebrospinal tract; mcSMT, multi-compartment spherical mean technique; MD, mean diffusivity; MDD, major depressive disorder; micro AX, microscopic axial diffusivity; MK, mean kurtosis; OCD, obsessive-compulsive disorder; SCZ, schizophrenia; SMT, spherical mean technique; radEAD, radial extra-axonal diffusivity; diffusivity; RK, radial kurtosis; Vcsf, cerebrospinal fluid fraction; WMTI, white matter tract integrity.

Although annual change in the cerebral peduncle showed the strongest associations with PRSs of AD and global WMM with ADHD, both global ($\bar{\beta }=0.015\;\pm \;0.012$) and regional ($\bar{\beta }=0.011\;\pm \;0.009$) WMM-PRS associations were nonsignificant after adjusting the α level for multiple comparisons (Figure 4A, B). Nevertheless, a highly brain region–specific pattern of associations between WMM ARoC and PRS was observed (Figure 4B), with the medial cerebral peduncle (Figure 4C) showing the strongest consistent associations with PRS and specifically associations with AD (|*β*_*max*_| = 0.053, $\left|\bar{\beta }\right|=0.014\;\pm \;0.013$) and MDD (|*β*_*max*_| = 0.051, $\left|\bar{\beta }\right|=0.014\;\pm \;0.013$) (Figure 4B, C). For all peduncle-PRS associations, see Supplemental Appendix W, indicating strongest ARoC-PRS [$\left|\bar{\beta }\right|=0.015$] associations but weaker nonreplicating associations for cross-sectional assessments [$\left|{\bar{\beta }}_{scan1}\right|=0.001$, $\left|{\bar{\beta }}_{scan2}\right|=0.001$, $\left|{\bar{\beta }}_{validation}\right|=0.001$]). Fornix, the most age-sensitive region, related strongest to ANX (|*β*_*max*_| = 0.011, $\left|\bar{\beta }\right|=\;0.006\;\pm \;0.003$) and OCD PRS (|*β*_*max*_| = 0.008, $\left|\bar{\beta }\right|=\;0.005\;\pm \;0.001$) (see Supplemental Appendix X).

**Figure 5:**
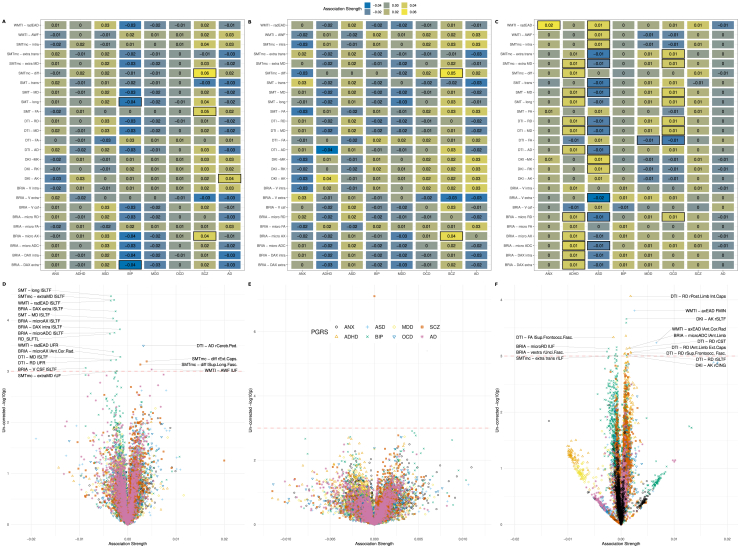
Cross-sectional associations between polygenic risk scores (PRSs) and white matter microstructure (WMM) in longitudinal and cross-sectional validation data. **(A)** PRS associations of globally averaged WMM for time point 1 in the longitudinal sample (*n* = 2329) and **(B)** for time point 2, respectively. **(C)** Global WMM-PRS associations for the cross-sectional validation sample (*n* = 31,056). Boxes indicate significance at an uncorrected α < 0.05. For simplicity, standardized regression coefficients with |β| < 0.005 were rounded down to β = 0. **(D)** PRS associations of regionally averaged WMM for time point 1 in the longitudinal sample and **(E)** for time point 2. **(F)** Regional associations for the cross-sectional validation sample. The dotted line in panels **(D–F)** indicates an uncorrected α < 0.001. Labels presenting the respective regions and metrics are supplied above this α threshold, and |*β*| > 0.001. All associations were adjusted for age, sex, age × sex as fixed effects, and site as random effect, and none of the associations survived the adjustment of the α level for multiple comparisons. An overview of the utilized diffusion approaches and entailed WMM metrics can be found in Supplemental Appendix B. AD, Alzheimer’s disease; ADC, apparent diffusion coefficient; ADHD, attention-deficit/hyperactivity disorder; AK, axial kurtosis; ANX, anxiety; ASD, autism spectrum disorder; AWF, axonal water fraction; axEAD, axial extra axonal diffusivity; BIP, bipolar disorder; BRIA, Bayesian rotational invariant approach; CSF, cerebrospinal spinal fluid; DAX extra, extra-axonal axial diffusivity; DAX intra, intra-axonal axial diffusivity; DTI - AD, diffusion tensor imaging - axial diffusivity; DKI, diffusion kurtosis imaging; FA, fractional anisotropy; FMIN, Forceps Minor; mcSMT, multi-compartment spherical mean technique; MD, mean diffusivity; MDD, major depressive disorder; micro AX, microscopic axial diffusivity; MK, mean kurtosis; OCD, obsessive-compulsive disorder; radEAD, radial extra-axonal diffusivity; rCING, right Cingulum; rCST, right cerebrospinal tract; RD, radial diffusivity; RK, radial kurtosis; rSLTF, right superior longitudinal temporal fasciculus; rUF, right unicate fasciculus; SCZ, schizophrenia; SMT, spherical mean technique; Vcsf, cerebrospinal fluid fraction; V extra, extra-axonal water fraction; V intra, intra-axonal water fraction; WMTI, white matter tract integrity.

Similarly, observing each time point separately, both global and regional WMM were nonsignificant after adjusting for multiple comparisons. However, WMM-PRS associations were highly similar at the 2 time points in the longitudinal sample, yet highlighted BIP and SCZ associations (considering *p*_*uncorrected*_ < .05) (Figure 5A, B) did not replicate in an independent cross-sectional validation sample of 31,056 UKB participants (Figure 5C). Noteworthy, WMM-PRS associations were strongest for AD ($\left|{\bar{\beta }}_{scan1}\right|=0.0013$, $\left|{\bar{\beta }}_{scan2}\right|=0.0012$, $\left|{\bar{\beta }}_{validation}\right|=0.0005$) and ANX ($\left|{\bar{\beta }}_{scan1}\right|=0.0010$, $\left|{\bar{\beta }}_{scan2}\right|=0.0012$, $\left|{\bar{\beta }}_{validation}\right|=0.0004$) in the longitudinal sample, but strongest for ADHD ($\left|{\bar{\beta }}_{scan1}\right|=0.0008$, $\left|{\bar{\beta }}_{scan2}\right|=0.0010$, $\left|{\bar{\beta }}_{validation}\right|=0.0010$) and ASD ($\left|{\bar{\beta }}_{scan1}\right|=0.0011$, $\left|{\bar{\beta }}_{scan2}\right|=0.0008$, $\left|{\bar{\beta }}_{validation}\right|=0.0010$) in the cross-sectional validation sample (Figure 5D, F).

Overall, ARoC-PRS associations (${\bar{\beta }}_{long}$ = 0.011 ± 0.009) were significantly stronger than cross-sectional effects, ${\bar{\beta }}_{scan1}$ = 0.001 ± 0.001, *d* = 0.028, 95% CI 0.004–0.051, *p* < .001, ${\bar{\beta }}_{scan2}$ = 0.001 ± 0.001, *d* = 0.026, 95% CI, (0.003–0.049), *p* < .001, ${\bar{\beta }}_{validation}$ = 0.0007 ± 0.001, *d* = 0.031, 95% CI (0.008, 0.054), *p* < .001 (see Supplemental Appendix X for distribution of effects).

## Discussion

We investigated WMM changes using longitudinal UKB diffusion data using a series of diffusion approaches and their associations with PRS. The comparison between 2 time points, with an average interscan interval of $\bar{\Delta }$ = 2.44 years, was performed at different spatial scales to localize the strongest aging effect and its correlations with other covariates. Unadjusted time point differences in global WMM metrics (Supplemental Appendix C) resembled closely the covariate-adjusted time point differences, as well as age effects (Supplemental Appendix Ea, b). These aging effects largely confirmed previous findings showing a global decrease of FA and the increase in axial, radial, and mean diffusivity for both intra- and extra-axonal space (3,6,39,57, 58, 59), whereas kurtosis metrics decrease with age (3,6). Notably, we provided evidence for the intra-axonal water fraction estimated by different diffusion approaches demonstrating age-related decrease, accompanied by opposite trends for the extra-axonal water fraction and the cerebrospinal fluid water fraction (also known as free water in other diffusion approaches).

We observed accelerated change in global and regional WMM at higher ages. Observed inconsistencies in age dependencies of the annual change in axonal diffusivity stand in contrast to a previous longitudinal study analyzing DTI data on the voxel level (35). Such difference in the localization of the increased acceleration and in findings on axial diffusivity might be driven by methodological variations: here, we focused on global and regional averages of annual WMM change. Yet, in contrast to the mentioned study (35), another longitudinal investigation also did not detect accelerated axial diffusivity changes (60). However, our study provides larger statistical power than previous longitudinal investigations on the rate of change in WMM (35,60) and is hence benchmarking global and regional increases in the rate of WM change at a higher age.

Regionwise investigations allowed for more differentiated results and presented larger aging effects compared with global WMM metrics (Figure 3 and Supplemental Appendix J). Region-level assessments further underlined the strong effect of age on WMM (3,6,39,59,60). Differences between unadjusted and adjusted associations gave more detail to dependencies of regional WMM age associations on sex and scanner site. Unadjusted associations outlined the cerebellar peduncle and the superior longitudinal temporal fasciculus as regions with the largest age association, and the corpus callosum and fornix as the statistically most significant age-associated regions. After corrections, the fornix was identified as the most significant and strongest age-associated region, which is congruent with previous cross-sectional findings on limbic WMM age associations (3,4,39,61). These WMM age associations also followed the described global pattern of FA metrics decreasing at higher ages, and axial, radial, and mean diffusivity metrics increasing during aging.

The observed regional fornix changes were moreover clearly delineated in the voxel-level analysis. Additionally, a more general pattern was observed: radial, axial, and mean diffusivity metrics increased in superior frontal areas but decreased in more posterior and inferior areas (Table 1 and Supplemental Appendix Y). While previous findings show consistent DTI axial diffusivity, radial diffusivity and mean diffusivity increases and FA decreases across the brain for global and tract measures throughout aging (59,62), or particularly in the brain stem (35), no study has yet outlined such differential, nonhomogeneous spatial patterns of WMM changes across the brain. Only one other study (63) revealed a similar pattern for DTI when examining cross-sectional axial diffusivity, radial diffusivity, and mean diffusivity tract–age associations in the UKB, and recent reviews highlight the frontal lobes as most susceptible to white matter deterioration (64). Potentially, our findings provide additional evidence for the “last-in-first-out” retrogenesis hypothesis, which states that brain areas that develop slowest (such as the prefrontal cortex) are more vulnerable to negative aging effects, such as degeneration (65). On the other hand, the observed WMM changes might simply map onto frontal GM areas that are most affected by normal aging processes instead of areas with higher evolutionary expansion (65). Yet, as the age dependence of frontal WM seems to be partly explained by cognitive ability (66) and cerebrovascular factors (67), frontal WM might be particularly interesting for further clinical dMRI examinations.

In accordance with our previous findings (3,39), we found that the fornix is highly sensitive to aging-related changes. The fornix—a C-shaped bundle of nerve-fibers that acts as a major output tract of the hippocampus—is a brain region implicated in various neurological and psychiatric disorders, such as mild cognitive decline (68), impairment (69,70), Parkinson’s disease (71), arguably AD (72, 73, 74, 75, 76), and bipolar disorder (77). Moreover, the genetic architecture of fornix WMM is related to various neurologic and psychiatric disorders (78). Bridging such genetics and imaging findings indicates that there are genetic underpinnings for the accelerated aging of the fornix and other regions, which might explain pathology development. While our findings render the fornix as a promising marker of aging, future studies need to explore this region as a potential therapeutic target.

We identified general patterns of WM changes when applying diffusion approaches at voxelwise scale, namely 1) global FA metric decreases; and 2) axial, radial, and mean diffusivity metrics increase in superior frontal brain regions but 3) decrease in posterior regions, the brain stem, and the cerebellum. FA (DTI-FA and BRIA-microFA) decreases suggest different potential biological processes such as a seizing myelination or cell death across the brain (with microFA adding information on the fiber orientation coherence) (79) (Table 1 and Supplemental Appendix Y) or axonal degradation (80,81) in combination with intra-axonal water fraction metric. The other diffusion metrics contain information on axial, radial, and mean diffusivity and coherently suggested WMM degeneration with increasing age in superior frontal lobes and potential cell swelling in posterior and subcortical regions where diffusivity decreases. This is congruent with multi-compartment metrics from BRIA, SMT, and WM tract integrity (see Supplemental Appendix Y). Potentially, the decreases in diffusivity metrics in posterior and subcortical regions depict compensatory mechanisms, accounting for the frontal lobe deterioration. Additional examination of diffusion properties (e.g., using tractography) leveraging both single and multi-shell dMRI might provide further insight into the differential developments of WM across the adult life span.

Finally, this study presents a comprehensive overview of WM association with PRSs of common psychiatric disorders and AD. Moreover, we differentiated between global and regional WM metrics at each time point and the ARoC, which led to different association patterns. However, the associations of both WM and annual WM change with PRSs were nonsignificant when correcting for multiple comparisons. Previous results demonstrate small associations between WM and PRS in the UKB for MDD (38), SCZ, BIP (82), and AD (37), as well as in the ABCD (Adolescent Brain Cognitive Development) Study (83). For the further highlighting of key associations, we considered uncorrected *p* values, examining the PRS-WM associations as suggestive for potential associations of WM and genetic risk.

Surprisingly, the global annual rate of WMM change was associated only with the ADHD PRS. WMM at each time point provided a more nuanced association pattern including ANX, OCD, and AD, similar to previous findings on AD PRS-WM associations (37). Additionally, similarities between ANX and OCD PRS associations with WMM may originate from large symptom overlap between these diagnoses (84). However, this association pattern was not replicated in an independent cross-sectional portion of the UKB, which instead outlined associations with ASD and BIP. Therefore, whether these differential association patterns speak to sample-specific gene-brain relationships or are simply noise due to a lack of statistical power (as a function of small effect sizes) requires follow-up with larger samples, more time points, and larger interscan intervals.

Observing relationships of PRS with both WMM and its ARoC in single regions highlight the brain stem, cerebral peduncle, and limbic system as potential PRS association targets. Importantly, PRS associations were orders of magnitude stronger for WMM rate of change than for cross-sectional metrics, which underlines the importance of examining the genetic underpinnings of WM in longitudinal data. Notably, areas outlined as most age sensitive (the fornix and cerebral peduncle) were also the strongest related in their annual change to ANX, ADHD, OCD, and SCZ-PRS. However, more longitudinal research is needed to validate the presented findings. Genetic overlaps between fornix WMM and the listed disorders (78), as well as the involvement of the cerebellum, which is connected with the cortex via the cerebral peduncles, in various psychiatric disorders gives additional insight into the role of genetic makeup for WMM development (85). Furthermore, the cerebral peduncles were particularly associated not only with AD, ADHD, and OCD PRSs but also with ANX, BIP, MDD, and SCZ PRSs. This spatially specific pattern of PRS associations emphasizes the usefulness of regional investigations due to highly spatially distributed influence of genetics. While the small effect sizes limit the inferences on WMM-PRS associations, the highlighted associations of PRS and WMM change are worthy of further investigation.

There are several limitations to be mentioned in the context of this study. First, the age range was limited to individuals older than 40 years, allowing only for generalizations across mid-to-late adulthood. Future studies should consider large samples to cover the whole life span, particularly when the objective is to investigate WM aging or to investigate generalizable associations of WM with genotypes and phenotypes. Second, the interscan interval was relatively short ($\bar{\Delta }$ = 2.44 years). Longer interscan intervals might reveal clearer information on accelerated WMM aging processes at different ages and their genetic underpinnings. Longer intervals are also useful to examine the relationship of cognitive decline and WMM. Third, we used a relatively homogeneous, nondiverse sample including nearly exclusively White UK citizens, which limits the generalizability beyond White northern Europeans and US Americans in midlife to older age. Additionally, although the sample size was larger than in previous longitudinal WMM investigations, power was still limited to find PRS associations (presenting small effects). The volunteer-based sampling of the UKB participants might additionally have introduced bias, reducing generalizability to the UK population (86). Yet, the imaging sample of the UKB shows an additional positive health bias (better physical and mental health) over the rest of the UKB sample (87), rendering this subsample as even less representative of the UK population. However, this might not necessarily be a disadvantage considering the objective of this study, which was to map WM change in healthy mid-to-late life adults and associate polygenic risk with the observed WM changes. Fourth, conservative corrections of the α level using Bonferroni corrections might have led to false negatives, especially for the small observed effects of PRSs on the annual change in regional WMM where many associations were explored. Finally, the fornix, the key region of the presented regional WMM changes, is a region susceptible to free water contamination due to its closeness to the cerebrospinal fluid (2) and has previously been suspected of partial volume effects (61). Hence, findings on this region need to be interpreted carefully.

### Conclusions

Our findings provide insight about short-term WM changes indicating degradation processes indicated by lower FA, kurtosis, intra-axonal water fraction, and higher diffusivity, free water, and extra-axonal water fraction. These changes are associated with the demyelination and structural disintegration across the adult brain as a consequence of aging without strong or detectable cognitive decline. Demyelination and WM features degradation primarily affect frontal brain regions, whereas posterior regions as well as brain stem and cerebellum show opposing trends. Further investigations should focus on fornix WMM changes throughout the life span to investigate health and disease outcomes, and the role in such of the genetic architecture of the cerebellar peduncle in such white matter changes and disease development.

## Supplementary Material

Key Resources Table
