# Supplementary material for: Distinct Longitudinal Brain White Matter Microstructure Changes and Associated Polygenic Risk of Common Psychiatric Disorders and Alzheimer’s Disease in the UK Biobank
Source: Biol Psychiatry Glob Open Sci. 2024 Apr 26;4(4):100323. doi: 10.1016/j.bpsgos.2024.100323 (PMC11313202; doi:10.1016/j.bpsgos.2024.100323)
Supplement: Supplement [file mmc2.pdf]

SUPPLEMENTARY INFORMATION

Distinct Longitudinal Brain White Matter Microstructure Changes and Associated Polygenic Risk of Common Psychiatric Disorders and Alzheimer's Disease in the UK Biobank

Korbmacher *et al.*

Appendices

Appendix A The utilized white matter quality control pipeline.

In brief, YTTRIUM[1] converts dMRI scalar metrics into 2D format, using a structural similarity[2, 3] extension of each scalar map to their mean image in order to create a 2D distribution of image and diffusion parameters. These quality assessments are based on a 2-step clustering algorithm applied to identify subjects located out of the main distribution. Additionally, rows including impossible values, such as diffusion coefficients d: $0 < d < 4$ $\mu\text{m}^2{\cdot}\text{ms}$, kurtosis values K $0 < K < 3$, and FA values $0 < FA < 1$ were excluded. QC of the mean skeleton values rendered $N = 622$ datasets of the BRIA metric extra-axonal radial diffusivity (DRADextra) as such impossible values, and examining regional and tract averages $N = 643$ of the WMTI metric axial extra-axonal diffusivity (axEAD). Due to the relatively large share of these outliers on the total sample, we excluded these metrics from the analyses.

Appendix B Description of white matter features by diffusion approaches.

Diffusion Approach	Metrics
Bayesian Rotationally Invariant Approach (BRIA)[4]	intra-axonal axial diffusivity (DAX intra)
	extra-axonal radial diffusivity (DRAD extra)*
	microscopic fractional anisotropy (micro FA)
	extra-axonal axial diffusivity (DAX extra)
	intra-axonal water fraction (V intra)
	extra-axonal water fraction (V extra)
	cerebrospinal fluid fraction (vCSF)
	microscopical axial diffusivity (micro AX)
	microscopic radial diffusivity (micro RD)
	microscopical apparent diffusion coefficient (micro ADC)
Diffusion Kurtosis Imaging (DKI)[5, 9]	mean kurtosis (MK)
	radial kurtosis (RK)
	axial kurtosis (AK)
Diffusion Tensor Imaging (DTI)[7]	fractional anisotropy (FA)
	axial diffusivity (AD)
	mean diffusivity (MD)
	radial diffusivity (RD)
Spherical Mean Technique (SMT)[8]	fractional anisotropy (SMT FA)
	mean diffusivity (SMT md)
	transverse diffusion coefficient (SMT trans)
	longitudinal diffusion coefficient (SMT long)
Multi-compartment Spherical Mean Technique (SMTmc)[10]	extra-neurite microscopic
	mean diffusivity (SMTmc extra md)
	extra-neurite transverse microscopic diffusivity
	(SMTmc extra trans)
	mc SMTdiffusion coefficient (SMT mcd)
	intra-neurite volume fraction (SMTmc intra)
White Matter Tract Integrity (WMTI)[9]	axonal water fraction (AWF)
	radial extra-axonal diffusivity (radEAD)
	axial extra-axonal diffusivity (axEAD)*

*Note that Drad extra and axEAD were excluded from the analyses as a significant portion of the produced metrics did not pass our quality control procedure[1].

Appendix C Effect sizes for unadjusted microstructure changes between time points.

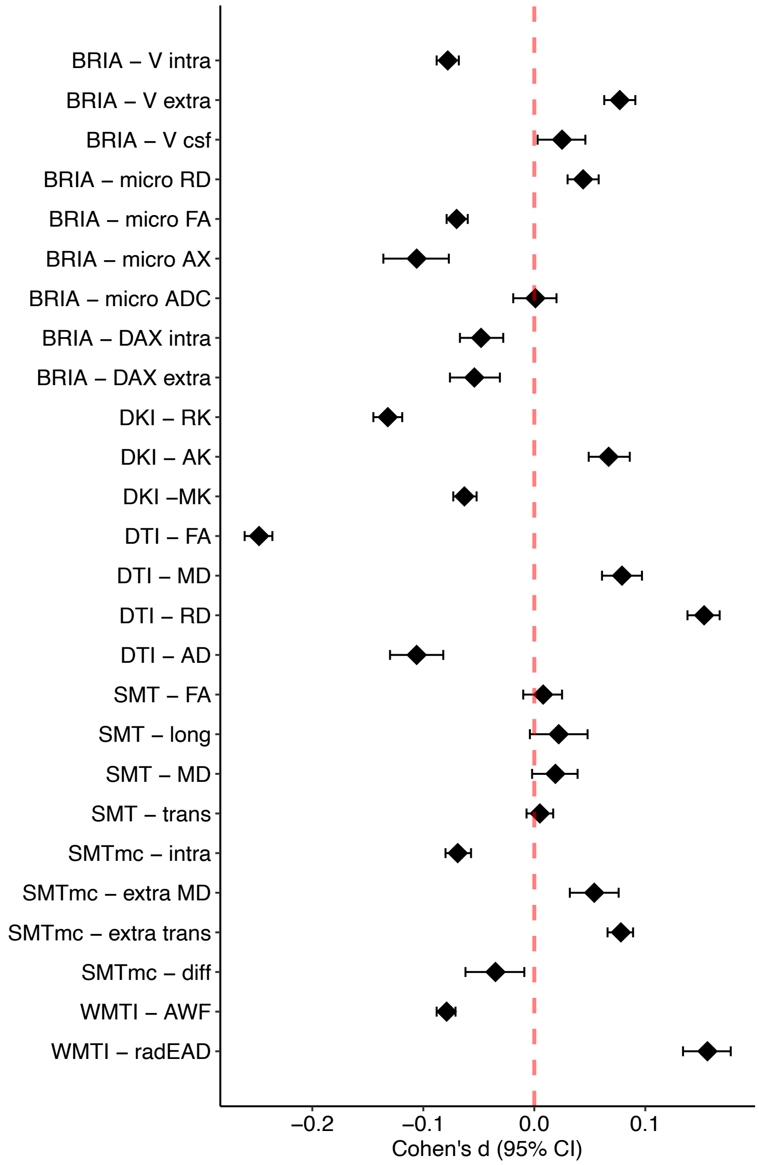

Appendix D Test statistics for time point differences for global diffusion metrics.

The table presents mean (M), standard deviation (SD) for each time point (TP), T-statistic (T), uncorrected p-value (p), Bonferroni-corrected p-value p_{Bonf}, and Cohen's d with 95% CI as effect size.

**Metric**	**M**$_{TP1}$	**SD**$_{TP1}$	**M**$_{TP2}$	**SD**$_{TP2}$	**T**	**p**	**p**$_{adj}$	**Cohens's d**	**L 95% CI**	**U 95% CI**
BRIA - V intra	0.499	0.027	0.497	0.028	16.051	<0.001	<0.001	-0.078	-0.088	-0.068
BRIA - V extra	0.414	0.025	0.416	0.026	-10.937	<0.001	<0.001	0.077	0.063	0.091
BRIA - V csf	0.087	0.008	0.087	0.009	-2.254	0.024	0.631	0.025	0.003	0.046
BRIA - micro RD	0.524	0.038	0.526	0.039	-6.166	<0.001	<0.001	0.044	0.03	0.058
BRIA - micro FA	0.64	0.019	0.638	0.02	13.977	<0.001	<0.001	-0.07	-0.079	-0.06
BRIA - micro AX	1.899	0.032	1.896	0.032	7.132	<0.001	<0.001	-0.106	-0.136	-0.077
BRIA - micro ADC	0.982	0.032	0.982	0.033	-0.082	0.935	1	0.001	-0.019	0.02
BRIA - DAX intra	2.196	0.035	2.194	0.036	4.825	<0.001	<0.001	-0.048	-0.067	-0.028
BRIA - DAX extra	1.199	0.024	1.198	0.025	4.747	<0.001	<0.001	-0.054	-0.076	-0.031
DKI - RK	1.467	0.072	1.458	0.074	19.808	<0.001	<0.001	-0.132	-0.145	-0.119
DKI - AK	0.777	0.021	0.778	0.022	-7.124	<0.001	<0.001	0.067	0.049	0.086
DKI -MK	1.058	0.038	1.056	0.039	11.5	<0.001	<0.001	-0.063	-0.073	-0.052
DTI - FA	0.463	0.018	0.458	0.019	40.372	<0.001	<0.001	-0.248	-0.261	-0.236
DTI - MD	0.886	0.028	0.888	0.029	-8.679	<0.001	<0.001	0.079	0.061	0.097
DTI - RD	0.64	0.031	0.645	0.033	-20.295	<0.001	<0.001	0.153	0.138	0.167
DTI - AD	1.377	0.028	1.374	0.028	8.761	<0.001	<0.001	-0.106	-0.13	-0.082
SMT - FA	0.935	0.007	0.935	0.007	-0.844	0.399	1	0.008	-0.01	0.025
SMT - long	0.003	0	0.003	0	-1.652	0.099	1	0.022	-0.004	0.048
SMT - MD	0.001	0	0.001	0	-1.787	0.074	1	0.019	-0.002	0.039
SMT - trans	0	0	0	0	-0.778	0.436	1	0.005	-0.007	0.017
SMTmc - intra	0.592	0.03	0.59	0.031	11.449	<0.001	<0.001	-0.069	-0.08	-0.057
SMTmc - extra MD	0.001	0	0.001	0	-4.781	<0.001	<0.001	0.054	0.032	0.076
SMTmc - extra trans	0.001	0	0.001	0	-12.994	<0.001	<0.001	0.078	0.066	0.089
SMTmc - diff	0.002	0	0.002	0	2.6	0.009	0.244	-0.035	-0.062	-0.009
WMTI - AWF	0.39	0.014	0.388	0.014	18.187	<0.001	<0.001	-0.079	-0.088	-0.071
WMTI - radEAD	0.973	0.034	0.978	0.036	-14.445	<0.001	<0.001	0.156	0.134	0.177

Appendix E Effects of age, time point, sex and the age-sex interaction on global diffusion metrics in LMER.

Standardized β values $\pm$ Standard Error indicating the fixed effects of (a) age, (b) time point, (c) sex, and (d) sex$\times$age on global diffusion metrics. The color indicates the metric's corresponding diffusion approach.

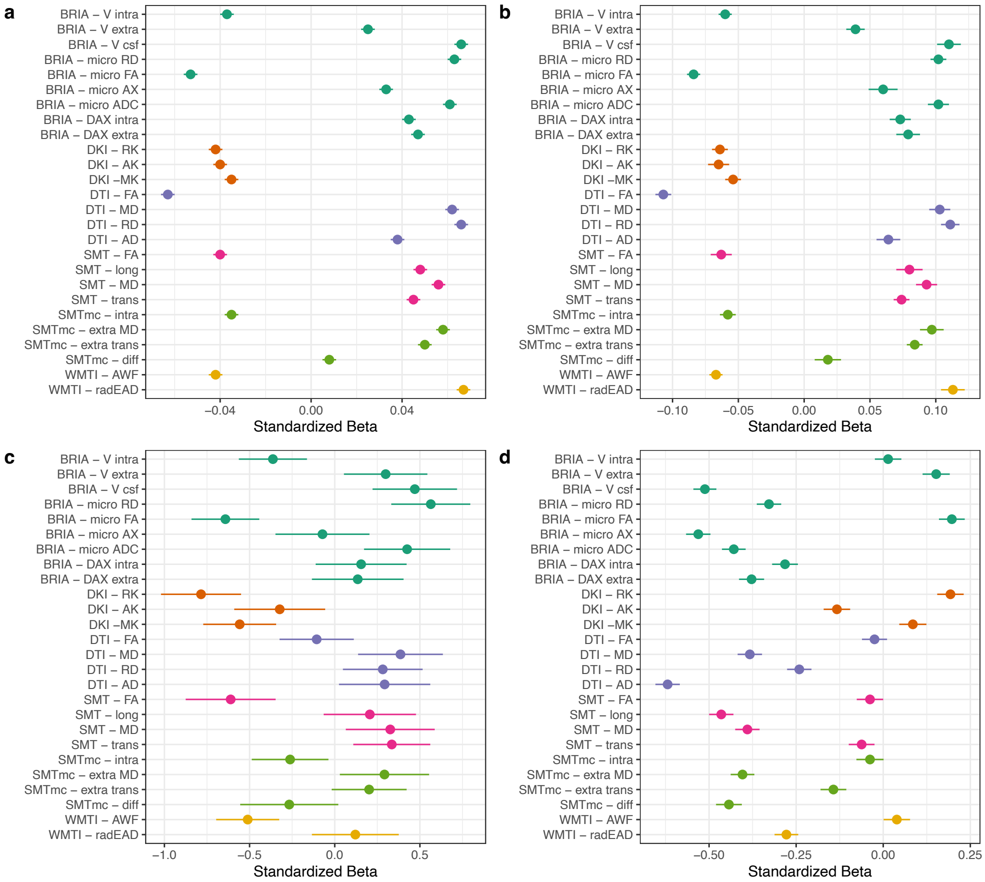

Appendix F Test statistics for the effect of age on global diffusion metrics in LMER.

β_{std} refers to the standardized slopes and SE to the standard error.

**Metric**	β_{std}	SE	p	$p_{Bonferroni}$
BRIA - V intra	-0.037	0.003	<.001	<.001
BRIA - V extra	0.025	0.003	<.001	<.001
BRIA - V csf	0.066	0.003	<.001	<.001
BRIA - micro RD	0.063	0.003	<.001	<.001
BRIA - micro FA	-0.053	0.003	<.001	<.001
BRIA - micro AX	0.033	0.003	<.001	<.001
BRIA - micro ADC	0.061	0.003	<.001	<.001
BRIA - DAX intra	0.043	0.003	<.001	<.001
BRIA - DAX extra	0.047	0.003	<.001	<.001
DKI - RK	-0.042	0.003	<.001	<.001
DKI - AK	-0.04	0.003	<.001	<.001
DKI -MK	-0.035	0.003	<.001	<.001
DTI - FA	-0.063	0.003	<.001	<.001
DTI - MD	0.062	0.003	<.001	<.001
DTI - RD	0.066	0.003	<.001	<.001
DTI - AD	0.038	0.003	<.001	<.001
SMT - FA	-0.04	0.003	<.001	<.001
SMT - long	0.048	0.003	<.001	<.001
SMT - MD	0.056	0.003	<.001	<.001
SMT - trans	0.045	0.003	<.001	<.001
SMTmc - intra	-0.035	0.003	<.001	<.001
SMTmc - extra MD	0.058	0.003	<.001	<.001
SMTmc - extra trans	0.05	0.003	<.001	<.001
SMTmc - diff	0.008	0.003	0.01	0.269
WMTI - AWF	-0.042	0.003	<.001	<.001
WMTI - radEAD	0.067	0.003	<.001	<.001

Appendix G The effect of time point on global diffusion metrics in mixed linear models.

**Metric**	β_{std}	SE	p	$p_{Bonferroni}$
BRIA - V intra	-0.06	0.005	<.001	<.001
BRIA - V extra	0.039	0.007	<.001	<.001
BRIA - V csf	0.11	0.009	<.001	<.001
BRIA - micro RD	0.102	0.006	<.001	<.001
BRIA - micro FA	-0.084	0.005	<.001	<.001
BRIA - micro AX	0.06	0.011	<.001	<.001
BRIA - micro ADC	0.102	0.008	<.001	<.001
BRIA - DAX intra	0.073	0.008	<.001	<.001
BRIA - DAX extra	0.079	0.009	<.001	<.001
DKI - RK	-0.064	0.006	<.001	<.001
DKI - AK	-0.065	0.008	<.001	<.001
DKI -MK	-0.054	0.006	<.001	<.001
DTI - FA	-0.107	0.006	<.001	<.001
DTI - MD	0.103	0.008	<.001	<.001
DTI - RD	0.111	0.007	<.001	<.001
DTI - AD	0.064	0.009	<.001	<.001
SMT - FA	-0.063	0.008	<.001	<.001
SMT - long	0.08	0.01	<.001	<.001
SMT - MD	0.093	0.008	<.001	<.001
SMT - trans	0.074	0.006	<.001	<.001
SMTmc - intra	-0.058	0.006	<.001	<.001
SMTmc - extra MD	0.097	0.009	<.001	<.001
SMTmc - extra trans	0.084	0.006	<.001	<.001
SMTmc - diff	0.018	0.01	0.082	1
WMTI - AWF	-0.067	0.005	<.001	<.001
WMTI - radEAD	0.113	0.009	<.001	<.001

Appendix H Mixed linear model diagnostics.

The following table shows the model diagnostics when predicting diffusion metrics from age, sex, age×sex, and time point, while accounting for the participants nested in scanner site as random intercept.

**Metric**	**AIC**	**BIC**	**Conditional R^2**	**Marginal R^2**
BRIA - V intra	7618.31	7677.58	0.97	0.07
BRIA - V extra	9655.94	9715.21	0.93	0.03
BRIA - V csf	11005.42	11064.69	0.84	0.28
BRIA - micro RD	9178.08	9237.35	0.93	0.21
BRIA - micro FA	7516.52	7575.80	0.96	0.14
BRIA - micro AX	13016.03	13075.30	0.70	0.13
BRIA - micro ADC	10798.61	10857.88	0.86	0.22
BRIA - DAX intra	11163.76	11223.03	0.87	0.11
BRIA - DAX extra	11683.87	11743.15	0.83	0.14
DKI - RK	9232.04	9291.32	0.94	0.09
DKI - AK	11036.27	11095.54	0.88	0.08
DKI -MK	8242.74	8302.02	0.96	0.06
DTI - FA	8388.40	8447.67	0.95	0.20
DTI - MD	10352.80	10412.07	0.89	0.22
DTI - RD	9350.35	9409.62	0.92	0.23

Appendix I The effect of sex on global diffusion metrics in LMER.

**Metric**	β_{std}	SE	p	$p_{Bonferroni}$
BRIA - V intra	-0.363	0.2	0.069	1
BRIA - V extra	0.299	0.245	0.222	1
BRIA - V csf	0.47	0.248	0.059	1
BRIA - micro RD	0.564	0.232	0.015	0.386
BRIA - micro FA	-0.642	0.199	0.001	0.034
BRIA - micro AX	-0.072	0.276	0.793	1
BRIA - micro ADC	0.425	0.253	0.093	1
BRIA - DAX intra	0.155	0.267	0.561	1
BRIA - DAX extra	0.135	0.269	0.616	1
DKI - RK	-0.785	0.235	0.001	0.022
DKI - AK	-0.323	0.267	0.227	1
DKI -MK	-0.558	0.214	0.009	0.237
DTI - FA	-0.106	0.218	0.626	1
DTI - MD	0.386	0.249	0.121	1
DTI - RD	0.282	0.234	0.229	1
DTI - AD	0.293	0.268	0.274	1
SMT - FA	-0.611	0.264	0.021	0.538
SMT - long	0.206	0.271	0.446	1
SMT - MD	0.326	0.261	0.211	1
SMT - trans	0.335	0.226	0.138	1
SMTmc - intra	-0.262	0.225	0.245	1
SMTmc - extra MD	0.292	0.262	0.265	1
SMTmc - extra trans	0.202	0.22	0.359	1
SMTmc - diff	-0.267	0.288	0.353	1
WMTI - AWF	-0.511	0.185	0.006	0.149
WMTI - radEAD	0.121	0.255	0.635	1

Appendix J The effect of the sex-age interaction on global diffusion metrics in LMER.

**Metric**	β_{std}	SE	p	$p_{Bonferroni}$
BRIA - V intra	0.014	0.038	0.708	1
BRIA - V extra	0.152	0.039	<0.001	0.003
BRIA - V csf	-0.512	0.033	<0.001	<0.001
BRIA - micro RD	-0.328	0.035	<0.001	<0.001
BRIA - micro FA	0.197	0.037	<0.001	<0.001
BRIA - micro AX	-0.531	0.035	<0.001	<0.001
BRIA - micro ADC	-0.429	0.034	<0.001	<0.001
BRIA - DAX intra	-0.282	0.037	<0.001	<0.001
BRIA - DAX extra	-0.378	0.036	<0.001	<0.001
DKI - RK	0.193	0.038	<0.001	<0.001
DKI - AK	-0.133	0.038	<0.001	0.012
DKI -MK	0.085	0.039	0.028	0.737
DTI - FA	-0.025	0.036	0.491	1
DTI - MD	-0.383	0.035	<0.001	<0.001
DTI - RD	-0.241	0.035	<0.001	<0.001
DTI - AD	-0.619	0.035	<0.001	<0.001
SMT - FA	-0.038	0.038	0.314	1
SMT - long	-0.465	0.035	<0.001	<0.001
SMT - MD	-0.39	0.035	<0.001	<0.001
SMT - trans	-0.062	0.037	0.1	1
SMTmc - intra	-0.038	0.039	0.327	1
SMTmc - extra MD	-0.404	0.034	<0.001	<0.001
SMTmc - extra trans	-0.143	0.037	<0.001	0.003
SMTmc - diff	-0.443	0.037	<0.001	<0.001
WMTI - AWF	0.039	0.038	0.302	1
WMTI - radEAD	-0.278	0.034	<0.001	<0.001

Appendix K The effect of the sex on regional WMM metrics.

Increases refer to higher WMM values for males compared to females and decrease to lower WMM values, respectively.

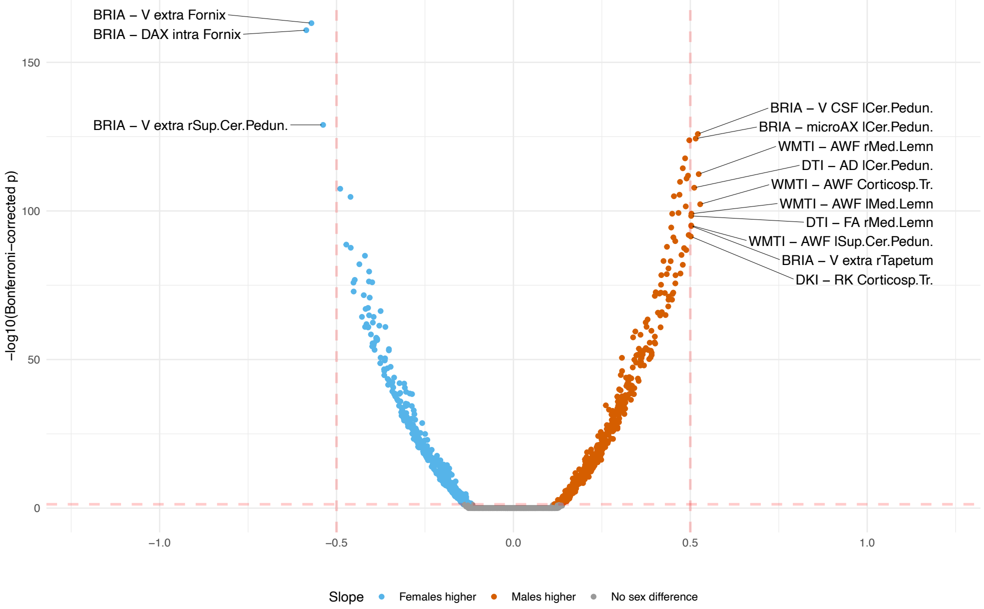

Appendix L The effect of the sex-age interaction on regional WMM metrics.

Increases refer for higher WMM values for males at higher ages compared to females and decrease to lower WMM values, respectively.

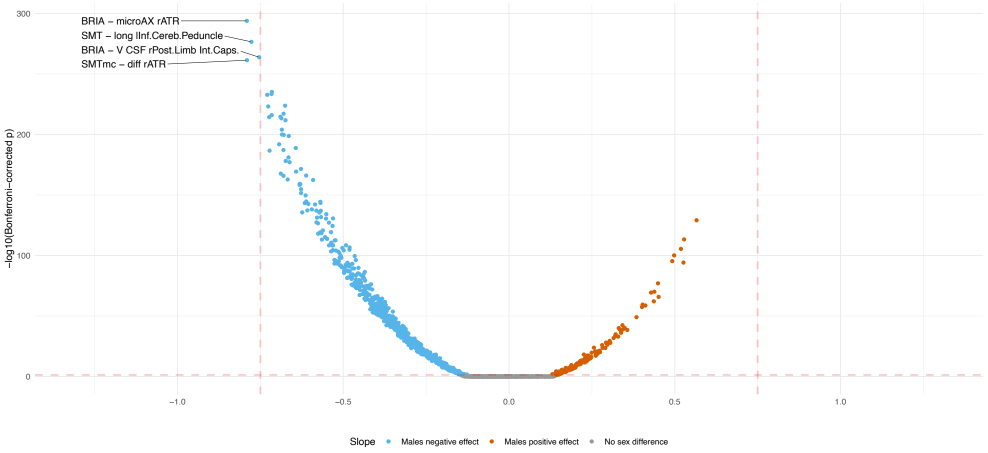

Appendix M Distribution of the effect of age on regional WMM metrics.

Estimating average slopes from all β-values results in the following meta-statistics for negative β-values (left side of 0): Mean = -0.21±0.13, Median = -0.23± 0.12. For positive β-values (right side of 0) the following averages were estimated: Mean = 0.25±0.13, Median = 0.27± 0.14.
Estimating average slopes from significant effects only (right panel) results in similar meta-statistics for negative β-values (left side of 0): Mean = -0.21±0.13, Median = -0.24± 0.12, as well as for positive β-values (right side of 0): Mean = 0.25±0.13, Median = 0.27± 0.15.

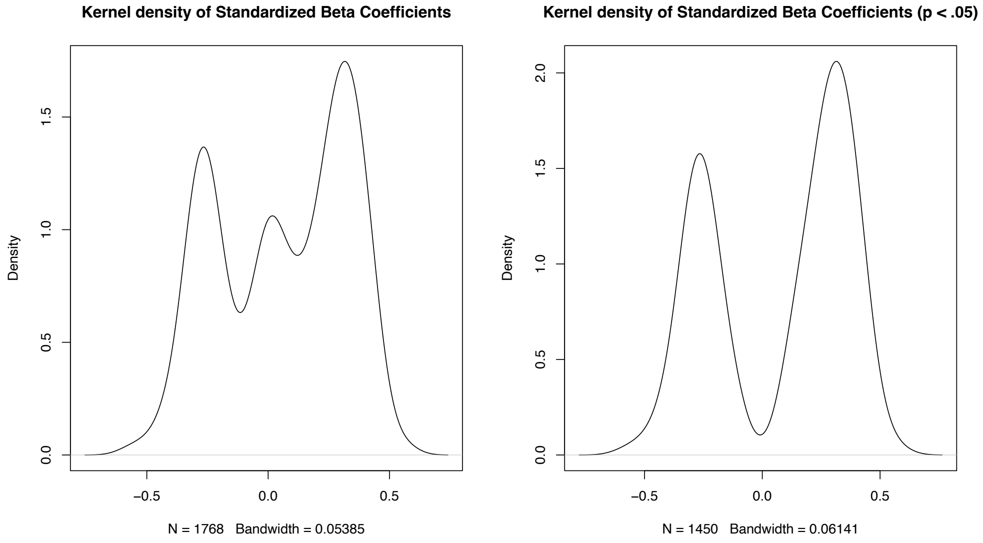

Appendix N Adjusted cross-sectional associations between global WMM and age at time point 1.

Age-WMM relationships were adjusted for sex, the sex-age interaction, and site with the linear and non-linear fit indicated.

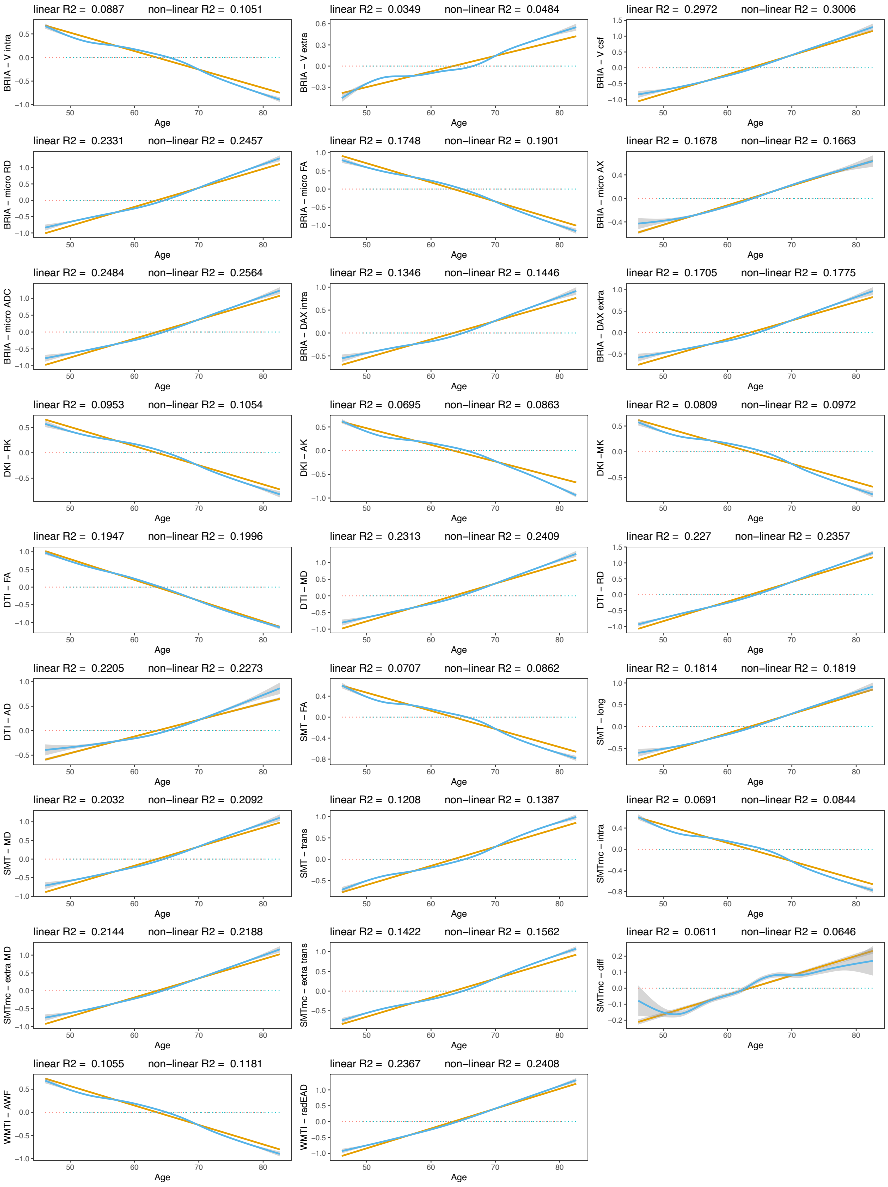

Appendix O Adjusted cross-sectional associations between global WMM and age at time point 2.

Age-WMM relationships were adjusted for sex, the sex-age interaction, and site with the linear and non-linear fit indicated.

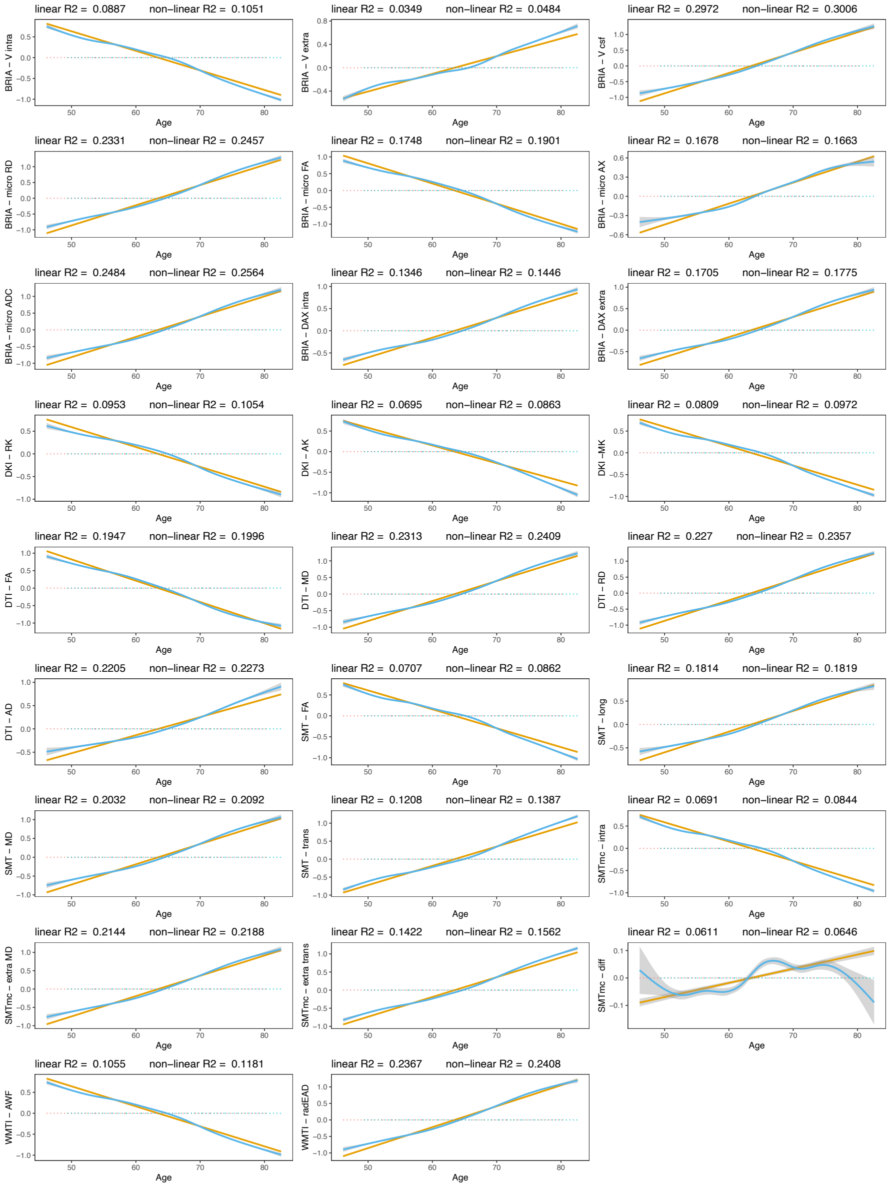

Appendix P Cognitive test differences between time points.

Pair matches refers to incorrectly solved pair matches (executed in 3 rounds). Digits refers to the maximum number of digits remembered; intelligence to fluid intelligence; memory to prospective memory; health to a rating of the own overall health; matrix RTs refer to matrix puzzle completion times; matrix correct to the number of correctly solved matrix puzzles; matrix viewed to the number of viewed matrix puzzles; tower correct to the number of correctly solved tower puzzles; and Sym/Dig matches to the number of correct symbol-digit matches.

**Metric**	M_{TP1}	SD_{TP1}	M_{TP2}	SD_{TP2}	*df*	*p*	p_{adj}	*d*	**L95%CI**	**U95%CI**
Inc. pair matches R1	0.297	0.758	0.289	0.716	2282	0.359	1	-0.026	-0.08	0.029
Inc. pair matches R2	3.344	2.52	3.225	2.586	2282	0.011	0.292	-0.067	-0.119	-0.015
Inc. pair matches R3	3.742	4.077	3.664	3.815	1037	0.049	1	-0.087	-0.174	0
Max. digits rem.	6.844	1.39	6.656	1.79	2184	<0.001	<0.001	-0.103	-0.149	-0.056
Fluid intel.	6.854	2.018	6.796	2.026	2219	0.9	1	0.002	-0.033	0.037
Prosp. mem.	1.045	0.363	1.048	0.354	2282	0.411	1	0.023	-0.031	0.077
Health self-rating	1.896	0.622	1.915	0.654	2654	0.123	1	0.027	-0.007	0.06
Matrix RT1	29.95	9.828	30.627	10.277	1488	0.02	0.526	0.07	0.011	0.129
Matrix RT2	32.601	12.741	33.117	24.712	1488	0.691	1	0.014	-0.053	0.08
Matrix RT3	58.936	26.351	61.716	30.574	1488	0.008	0.201	0.082	0.022	0.142
Matrix RT4	67.765	35.483	68.263	35.013	1488	0.778	1	0.009	-0.053	0.07
Matrix RT5	43.224	20.951	42.608	19.722	1488	0.124	1	-0.044	-0.1	0.012
Matrix RT6	96.575	53.267	95.419	52.752	1488	0.053	1	-0.058	-0.118	0.001
Matrix RT7	136.815	66.333	137.116	67.855	1488	0.987	1	0	-0.058	0.059
Matrix RT8	93.788	43.243	97.961	48.273	1487	0	0.011	0.097	0.043	0.151
Matrix RT9	173.672	102.302	173.778	98.117	1475	0.802	1	0.008	-0.053	0.068
Matrix RT10	92.188	46.633	92.784	48.648	1456	0.42	1	0.025	-0.036	0.086
Matrix RT11	203.247	108.918	192.572	106.136	1329	0.007	0.179	-0.087	-0.15	-0.024
Matrix RT12	189.715	90.756	178.99	87.538	1077	0	0.007	-0.131	-0.201	-0.06
Matrix RT13	127.154	72.968	122.417	72.97	865	0.112	1	-0.066	-0.147	0.015
Matrix RT14	164.675	75.616	155.445	73.219	576	0.204	1	-0.064	-0.164	0.035
Matrix RT15	134.735	70.439	128.53	66.241	350	0.278	1	-0.065	-0.182	0.052
Correct matrix puzzles	8.355	2.015	8.338	2.046	1488	0.618	1	0.012	-0.036	0.061
Viewed matrix puzzles	13.911	1.359	13.967	1.361	1488	0.151	1	0.036	-0.013	0.085
Correct tower puzzles	10.38	3.118	10.522	3.156	1473	0	0.002	0.105	0.053	0.156
Correct sym.-dig. matches	20.099	4.964	19.938	5.256	1472	0.764	1	0.007	-0.038	0.052

Appendix Q Distribution of the relationship between age and the absolute annual rate of white matter microstructure change.

The Figure shows the distribution of adjusted —ARoC—-age associations across brain regions ($\bar{\beta}_{padj<0.05} = 0.013 \pm 0.005$). Mean associations were smaller when including non-significant associations ($\bar{\beta}_{all} = 0.007 \pm 0.005$).

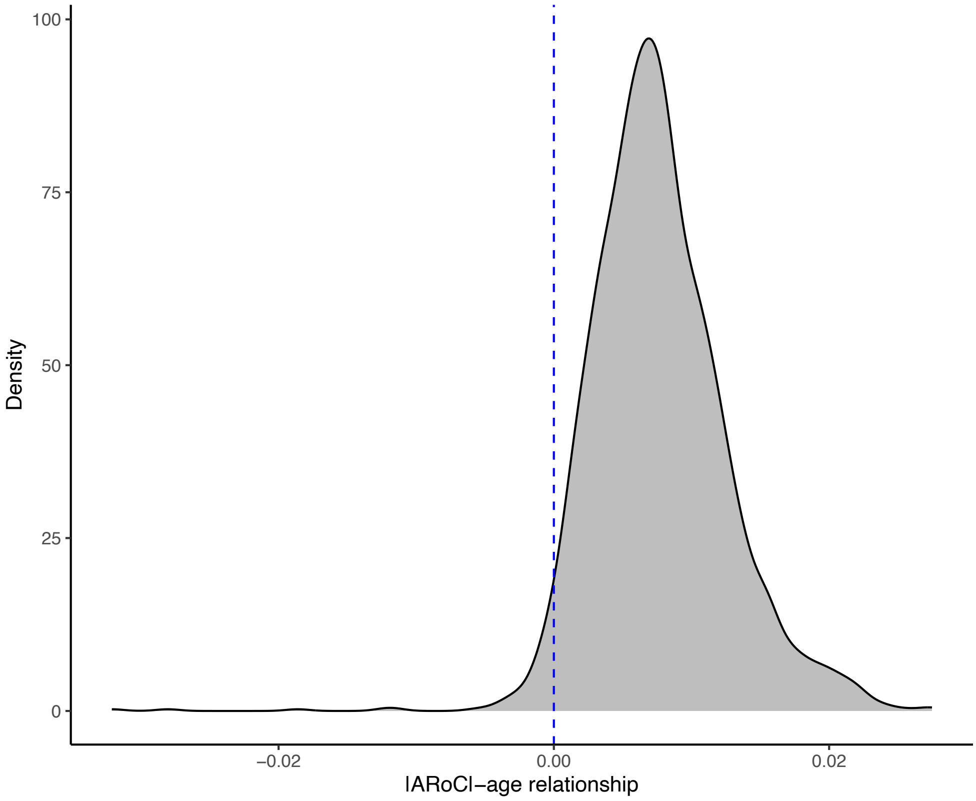

Appendix R Age-stratified annual rate of WMM change group-comparisons.

Table R1: Age-stratified annual rate of WMM change group-comparisons

Diffusion Metric	**Group 2**	**Group 2**	**Cohen's d**	$\mathbf{n}_1$	$\mathbf{n}_2$	$\mathbf{CI}_{low}$	$\mathbf{CI}_{high}$	**Magnitude**	$\mathbf{p}_{adj}$
BRIA - v intra	46-55	55-65	0.65	525	1157	0.54	0.78	moderate	<.001
BRIA - v intra	46-55	65-75	1.31	525	905	1.18	1.43	large	<.001
BRIA - v intra	46-55	75-81	1.78	525	91	1.54	2.06	large	<.001
BRIA - v intra	55-65	65-75	0.80	1157	905	0.71	0.9	large	<.001
BRIA - v intra	55-65	75-81	1.41	1157	91	1.14	1.73	large	<.001
BRIA - v intra	65-75	75-81	0.78	905	91	0.52	1.05	moderate	<.001
BRIA – v extra	46-55	55-65	-0.73	525	1157	-0.86	-0.6	moderate	<.001
BRIA – v extra	46-55	65-75	-1.44	525	905	-1.57	-1.31	large	<.001
BRIA – v extra	46-55	75-81	-1.88	525	91	-2.2	-1.62	large	<.001
BRIA – v extra	55-65	65-75	-0.94	1157	905	-1.04	-0.85	large	<.001
BRIA – v extra	55-65	75-81	-1.53	1157	91	-1.88	-1.26	large	<.001
BRIA – v extra	65-75	75-81	-0.83	905	91	-1.12	-0.58	large	<.001
BRIA – v CSF	46-55	55-65	-0.30	525	1157	-0.41	-0.19	small	<.001
BRIA – v CSF	46-55	65-75	-0.51	525	905	-0.62	-0.39	moderate	<.001
BRIA – v CSF	46-55	75-81	-0.71	525	91	-0.92	-0.5	moderate	<.001
BRIA – v CSF	55-65	65-75	-0.20	1157	905	-0.29	-0.12	small	<.001
BRIA – v CSF	55-65	75-81	-0.41	1157	91	-0.61	-0.2	small	0.001

Continued on next page

Table R1: Age-stratified annual rate of WMM change group-comparisons (Continued)

BRIA – v CSF	65-75	75-81	-0.21	905	91	-0.43	0.01	small	0.064
BRIA – micro RD	46-55	55-65	-0.45	525	1157	-0.55	-0.35	small	<.001
BRIA – micro RD	46-55	65-75	-0.85	525	905	-0.98	-0.74	large	<.001
BRIA – micro RD	46-55	75-81	-1.25	525	91	-1.5	-1.02	large	<.001
BRIA – micro RD	55-65	65-75	-0.42	1157	905	-0.51	-0.33	small	<.001
BRIA – micro RD	55-65	75-81	-0.87	1157	91	-1.13	-0.66	large	<.001
BRIA – micro RD	65-75	75-81	-0.48	905	91	-0.72	-0.26	small	<.001
BRIA – micro FA	46-55	55-65	0.56	525	1157	0.45	0.67	moderate	<.001
BRIA – micro FA	46-55	65-75	1.11	525	905	0.99	1.24	large	<.001
BRIA – micro FA	46-55	75-81	1.58	525	91	1.35	1.84	large	<.001
BRIA – micro FA	55-65	65-75	0.63	1157	905	0.55	0.72	moderate	<.001
BRIA – micro FA	55-65	75-81	1.21	1157	91	0.96	1.49	large	<.001
BRIA – micro FA	65-75	75-81	0.68	905	91	0.44	0.92	moderate	<.001
BRIA – micro AX	46-55	55-65	-0.06	525	1157	-0.16	0.04	negligible	0.478
BRIA – micro AX	46-55	65-75	0.04	525	905	-0.07	0.14	negligible	0.519
BRIA – micro AX	46-55	75-81	0.20	525	91	-0.05	0.43	small	0.332
BRIA – micro AX	55-65	65-75	0.09	1157	905	0.01	0.19	negligible	0.170
BRIA – micro AX	55-65	75-81	0.26	1157	91	0.04	0.48	small	0.121
BRIA – micro AX	65-75	75-81	0.16	905	91	-0.04	0.39	negligible	0.426
BRIA – micro ADC	46-55	55-65	-0.32	525	1157	-0.44	-0.22	small	<.001
BRIA – micro ADC	46-55	65-75	-0.55	525	905	-0.66	-0.43	moderate	<.001

Continued on next page

Table R1: Age-stratified annual rate of WMM change group-comparisons (Continued)

BRIA – micro ADC	46-55	75-81	-0.80	525	91	-1.03	-0.58	moderate	<.001
BRIA – micro ADC	55-65	65-75	-0.23	1157	905	-0.32	-0.14	small	<.001
BRIA – micro ADC	55-65	75-81	-0.48	1157	91	-0.7	-0.28	small	<.001
BRIA – micro ADC	65-75	75-81	-0.26	905	91	-0.48	-0.04	small	0.023
BRIA – DAX intra	46-55	55-65	-0.30	525	1157	-0.41	-0.2	small	<.001
BRIA – DAX intra	46-55	65-75	-0.51	525	905	-0.62	-0.4	moderate	<.001
BRIA – DAX intra	46-55	75-81	-0.74	525	91	-0.96	-0.52	moderate	<.001
BRIA – DAX intra	55-65	65-75	-0.20	1157	905	-0.29	-0.11	small	<.001
BRIA – DAX intra	55-65	75-81	-0.44	1157	91	-0.67	-0.23	small	<.001
BRIA – DAX intra	65-75	75-81	-0.24	905	91	-0.47	-0.02	small	0.033
BRIA – DAX extra	46-55	55-65	-0.26	525	1157	-0.36	-0.15	small	<.001
BRIA – DAX extra	46-55	65-75	-0.40	525	905	-0.51	-0.29	small	<.001
BRIA – DAX extra	46-55	75-81	-0.56	525	91	-0.78	-0.33	moderate	<.001
BRIA – DAX extra	55-65	65-75	-0.15	1157	905	-0.24	-0.05	negligible	0.003
BRIA – DAX extra	55-65	75-81	-0.31	1157	91	-0.53	-0.09	small	0.012
BRIA – DAX extra	65-75	75-81	-0.16	905	91	-0.39	0.06	negligible	0.145
DKI – RK	46-55	55-65	0.59	525	1157	0.48	0.7	moderate	<.001
DKI – RK	46-55	65-75	1.34	525	905	1.24	1.45	large	<.001
DKI – RK	46-55	75-81	1.90	525	91	1.69	2.17	large	<.001
DKI – RK	55-65	65-75	1.05	1157	905	0.98	1.14	large	<.001
DKI – RK	55-65	75-81	1.73	1157	91	1.44	2.05	large	<.001

Continued on next page

Table R1: Age-stratified annual rate of WMM change group-comparisons (Continued)

DKI – RK	65-75	75-81	0.98	905	91	0.71	1.29	large	<.001
DKI – AK	46-55	55-65	0.41	525	1157	0.31	0.51	small	<.001
DKI – AK	46-55	65-75	0.75	525	905	0.64	0.87	moderate	<.001
DKI – AK	46-55	75-81	1.12	525	91	0.88	1.36	large	<.001
DKI – AK	55-65	65-75	0.35	1157	905	0.27	0.44	small	<.001
DKI – AK	55-65	75-81	0.76	1157	91	0.54	0.99	moderate	<.001
DKI – AK	65-75	75-81	0.44	905	91	0.23	0.67	small	<.001
DKI – MK	46-55	55-65	0.61	525	1157	0.5	0.72	moderate	<.001
DKI – MK	46-55	65-75	1.22	525	905	1.09	1.34	large	<.001
DKI – MK	46-55	75-81	1.69	525	91	1.45	1.97	large	<.001
DKI – MK	55-65	65-75	0.72	1157	905	0.64	0.81	moderate	<.001
DKI – MK	55-65	75-81	1.32	1157	91	1.07	1.6	large	<.001
DKI – MK	65-75	75-81	0.74	905	91	0.51	0.99	moderate	<.001
DTI – FA	46-55	55-65	0.23	525	1157	0.11	0.34	small	<.001
DTI – FA	46-55	65-75	0.66	525	905	0.56	0.77	moderate	<.001
DTI – FA	46-55	75-81	1.00	525	91	0.74	1.29	large	<.001
DTI – FA	55-65	65-75	0.46	1157	905	0.36	0.55	small	<.001
DTI – FA	55-65	75-81	0.82	1157	91	0.58	1.1	large	<.001
DTI – FA	65-75	75-81	0.40	905	91	0.17	0.66	small	0.001
DTI – MD	46-55	55-65	-0.43	525	1157	-0.54	-0.33	small	<.001
DTI – MD	46-55	65-75	-0.79	525	905	-0.91	-0.67	moderate	<.001

Continued on next page

Table R1: Age-stratified annual rate of WMM change group-comparisons (Continued)

DTI – MD	46-55	75-81	-1.15	525	91	-1.41	-0.94	large	<.001
DTI – MD	55-65	65-75	-0.38	1157	905	-0.46	-0.29	small	<.001
DTI – MD	55-65	75-81	-0.77	1157	91	-1	-0.56	moderate	<.001
DTI – MD	65-75	75-81	-0.40	905	91	-0.62	-0.17	small	0.001
DTI – RD	46-55	55-65	-0.74	525	1157	-0.85	-0.63	moderate	<.001
DTI – RD	46-55	65-75	-1.47	525	905	-1.59	-1.36	large	<.001
DTI – RD	46-55	75-81	-1.92	525	91	-2.27	-1.63	large	<.001
DTI – RD	55-65	65-75	-0.93	1157	905	-1.04	-0.85	large	<.001
DTI – RD	55-65	75-81	-1.53	1157	91	-1.88	-1.24	large	<.001
DTI – RD	65-75	75-81	-0.76	905	91	-1.04	-0.52	moderate	<.001
DTI – AD	46-55	55-65	-0.22	525	1157	-0.33	-0.11	small	<.001
DTI – AD	46-55	65-75	-0.32	525	905	-0.43	-0.2	small	<.001
DTI – AD	46-55	75-81	-0.43	525	91	-0.67	-0.2	small	0.001
DTI – AD	55-65	65-75	-0.09	1157	905	-0.19	-0.0066	negligible	0.109
DTI – AD	55-65	75-81	-0.21	1157	91	-0.42	0.007	small	0.114
DTI – AD	65-75	75-81	-0.12	905	91	-0.35	0.08	negligible	0.289
SMT – FA	46-55	55-65	0.53	525	1157	0.43	0.64	moderate	<.001
SMT – FA	46-55	65-75	1.06	525	905	0.94	1.19	large	<.001
SMT – FA	46-55	75-81	1.51	525	91	1.28	1.77	large	<.001
SMT – FA	55-65	65-75	0.59	1157	905	0.5	0.68	moderate	<.001
SMT – FA	55-65	75-81	1.15	1157	91	0.93	1.41	large	<.001

Continued on next page

Table R1: Age-stratified annual rate of WMM change group-comparisons (Continued)

SMT – FA	65-75	75-81	0.66	905	91	0.43	0.91	moderate	<.001
SMT – long	46-55	55-65	-0.15	525	1157	-0.26	-0.04	negligible	0.021
SMT – long	46-55	65-75	-0.16	525	905	-0.27	-0.05	negligible	0.018
SMT – long	46-55	75-81	-0.11	525	91	-0.35	0.1	negligible	1.000
SMT – long	55-65	65-75	-0.01	1157	905	-0.11	0.07	negligible	1.000
SMT – long	55-65	75-81	0.04	1157	91	-0.19	0.25	negligible	1.000
SMT – long	65-75	75-81	0.05	905	91	-0.18	0.3	negligible	1.000
SMT – MD	46-55	55-65	-0.28	525	1157	-0.39	-0.18	small	<.001
SMT – MD	46-55	65-75	-0.46	525	905	-0.57	-0.35	small	<.001
SMT – MD	46-55	75-81	-0.64	525	91	-0.87	-0.42	moderate	<.001
SMT – MD	55-65	65-75	-0.18	1157	905	-0.26	-0.09	negligible	<.001
SMT – MD	55-65	75-81	-0.36	1157	91	-0.58	-0.15	small	0.003
SMT – MD	65-75	75-81	-0.19	905	91	-0.42	0.02	negligible	0.096
SMT – trans	46-55	55-65	-0.49	525	1157	-0.6	-0.38	small	<.001
SMT – trans	46-55	65-75	-0.93	525	905	-1.06	-0.82	large	<.001
SMT – trans	46-55	75-81	-1.36	525	91	-1.61	-1.14	large	<.001
SMT – trans	55-65	65-75	-0.47	1157	905	-0.56	-0.39	small	<.001
SMT – trans	55-65	75-81	-0.97	1157	91	-1.23	-0.74	large	<.001
SMT – trans	65-75	75-81	-0.55	905	91	-0.8	-0.32	moderate	<.001
SMTmc – intra	46-55	55-65	0.70	525	1157	0.6	0.81	moderate	<.001
SMTmc – intra	46-55	65-75	1.36	525	905	1.23	1.49	large	<.001

Continued on next page

Table R1: Age-stratified annual rate of WMM change group-comparisons (Continued)

SMTmc – intra	46-55	75-81	1.82	525	91	1.56	2.14	large	<.001
SMTmc – intra	55-65	65-75	0.82	1157	905	0.73	0.9	large	<.001
SMTmc – intra	55-65	75-81	1.42	1157	91	1.15	1.75	large	<.001
SMTmc – intra	65-75	75-81	0.76	905	91	0.5	1.03	moderate	<.001
SMTmc – extra MD	46-55	55-65	-0.32	525	1157	-0.43	-0.21	small	<.001
SMTmc – extra MD	46-55	65-75	-0.55	525	905	-0.67	-0.44	moderate	<.001
SMTmc – extra MD	46-55	75-81	-0.77	525	91	-1	-0.55	moderate	<.001
SMTmc – extra MD	55-65	65-75	-0.23	1157	905	-0.32	-0.14	small	<.001
SMTmc – extra MD	55-65	75-81	-0.46	1157	91	-0.67	-0.24	small	<.001
SMTmc – extra MD	65-75	75-81	-0.23	905	91	-0.45	-0.01	small	0.041
SMTmc – extra trans	46-55	55-65	-0.61	525	1157	-0.72	-0.49	moderate	<.001
SMTmc – extra trans	46-55	65-75	-1.16	525	905	-1.28	-1.05	large	<.001
SMTmc – extra trans	46-55	75-81	-1.63	525	91	-1.92	-1.36	large	<.001
SMTmc – extra trans	55-65	65-75	-0.64	1157	905	-0.73	-0.55	moderate	<.001
SMTmc – extra trans	55-65	75-81	-1.20	1157	91	-1.49	-0.95	large	<.001
SMTmc – extra trans	65-75	75-81	-0.64	905	91	-0.9	-0.4	moderate	<.001
SMTmc – diff	46-55	55-65	0.61	525	1157	0.51	0.72	moderate	<.001
SMTmc – diff	46-55	65-75	1.23	525	905	1.12	1.34	large	<.001
SMTmc – diff	46-55	75-81	1.73	525	91	1.44	2.06	large	<.001
SMTmc – diff	55-65	65-75	0.78	1157	905	0.68	0.87	moderate	<.001
SMTmc – diff	55-65	75-81	1.40	1157	91	1.1	1.79	large	<.001

Continued on next page

Table R1: Age-stratified annual rate of WMM change group-comparisons (Continued)

SMTmc – diff	65-75	75-81	0.73	905	91	0.47	1.01	moderate	<.001
WMTI – AWF	46-55	55-65	0.55	525	1157	0.43	0.66	moderate	<.001
WMTI – AWF	46-55	65-75	1.11	525	905	0.99	1.24	large	<.001
WMTI – AWF	46-55	75-81	1.58	525	91	1.38	1.84	large	<.001
WMTI – AWF	55-65	65-75	0.65	1157	905	0.56	0.74	moderate	<.001
WMTI – AWF	55-65	75-81	1.24	1157	91	1.02	1.49	large	<.001
WMTI – AWF	65-75	75-81	0.72	905	91	0.51	0.97	moderate	<.001
WMTI – radEAD	46-55	55-65	-0.58	525	1157	-0.7	-0.47	moderate	<.001
WMTI – radEAD	46-55	65-75	-1.16	525	905	-1.28	-1.04	large	<.001
WMTI – radEAD	46-55	75-81	-1.52	525	91	-1.79	-1.27	large	<.001
WMTI – radEAD	55-65	65-75	-0.64	1157	905	-0.73	-0.56	moderate	<.001
WMTI – radEAD	55-65	75-81	-1.09	1157	91	-1.38	-0.87	large	<.001
WMTI – radEAD	65-75	75-81	-0.52	905	91	-0.77	-0.28	moderate	<.001

Appendix S Adjusted annual rate of WMM change throughout ages.

WMM are standardised for comparability without mean centering for a better understanding of onset values. Non-linear curves were fitted, using splines of generalized additive models, and Pearson's correlation coefficients were estimated (top left in each plot). We present uncorrected p-values, which were significant at the Bonferroni-corrected α ¡ $0.05/26 = 0.0019$. Additionally, we estimated associations between $|ARoC|$ and age (to meaningfully estimate meta-statistics), showing a higher $|ARoC|$ at higher ages ($\bar{\beta}_{sig} = 0.012 \pm 0.003$), with a slightly lower average slopes when considering non-significant age-$|ARoC|$ associations ($\bar{\beta}_{all} = 0.008 \pm 0.004$)

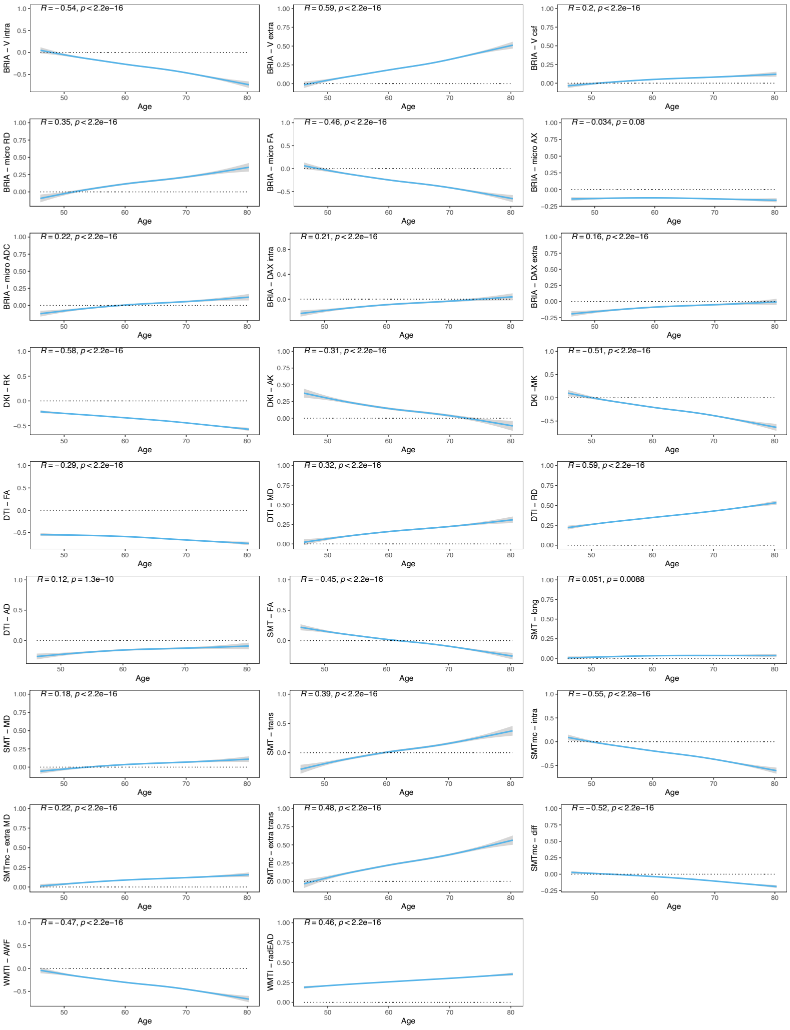

Appendix T Unadjusted annual rate of WMM change throughout ages.

WMM are standardised for comparability without mean centering for a better understanding of onset values. Red lines were implemented as visual aid.

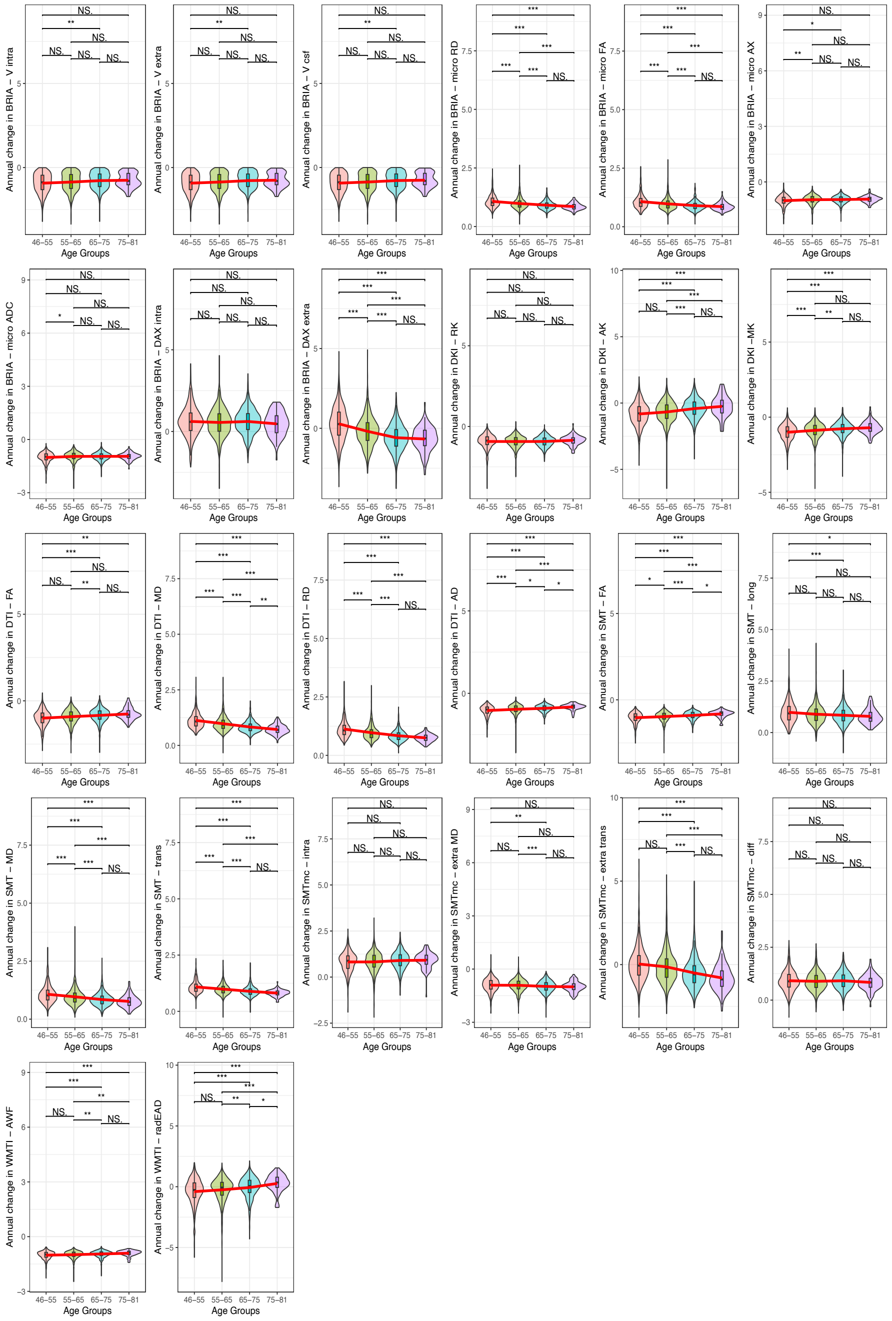

Appendix U Corrected sex- and age-stratified annual rate of WMM change.

WMM are standardised for comparability (including mean centering). Red colors indicate females and blue colors males.

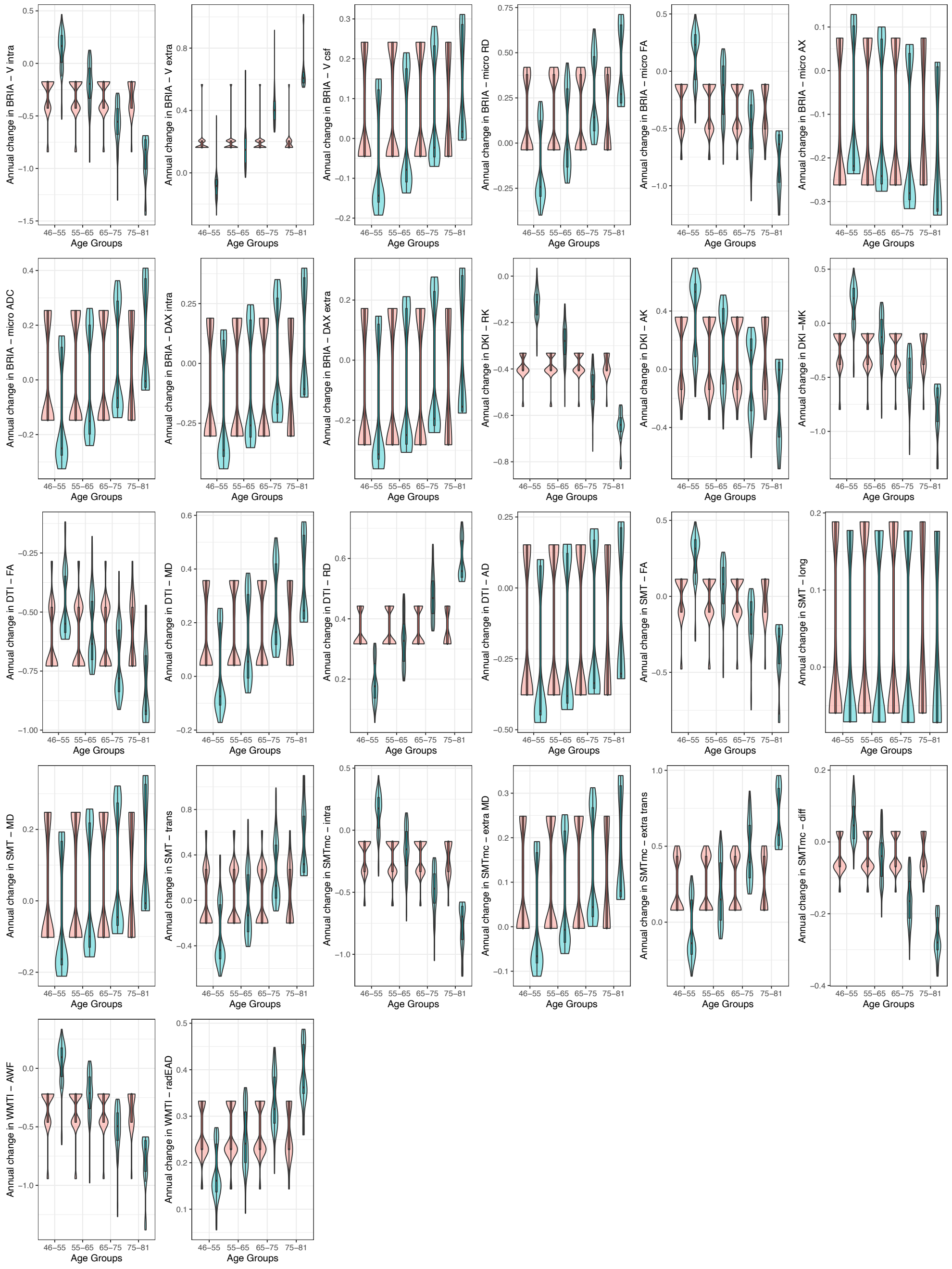

Appendix V Regional associations between WMM change in the Cerebral Peduncle and PGRS.

Panel (a) presents the associations between PGRS and WMM change. Panel (b) presents the regional associations between PGRS and cross-sectional regional WMM at time point one. Panel (c) presents the cross-sectional regional WMM associations with PGRS at time point two. Panel (d) presents cross-sectional regional associations between WMM and PGRS for the validation sample. Boxes the statistical significance at an uncorrected $\alpha < 0.05$. colors indicate the association strength (standardized β-coefficients). *Note:* All associations were adjusted for age, sex, the $age \times sex$ interaction, and site. None of the presented associations survived the adjustment of the α-level for multiple comparisons.

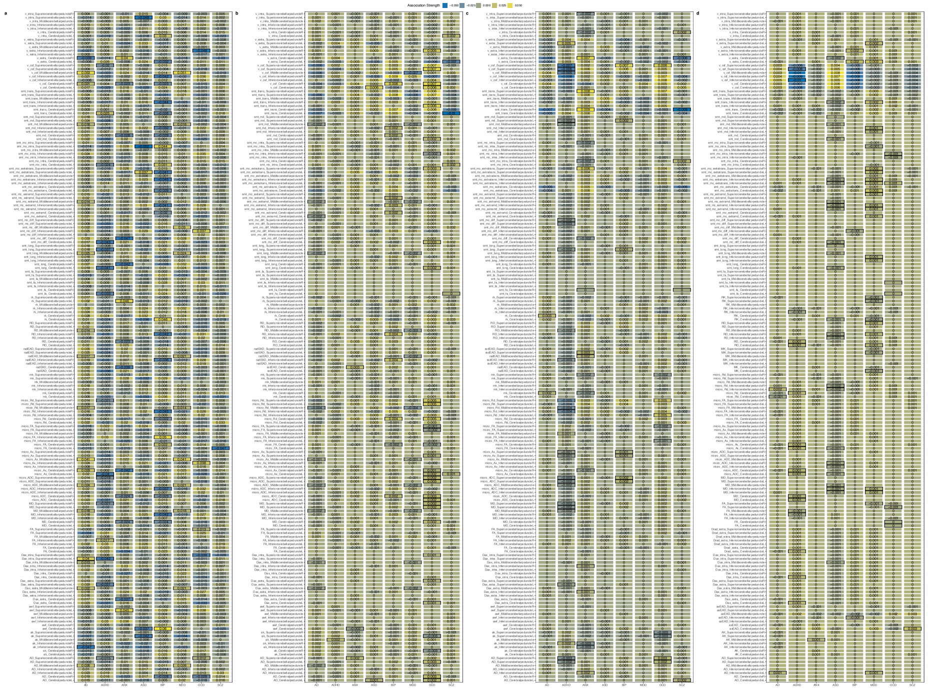

Appendix W Regional associations between WMM change in the Fornix and PGRS.

Panel (a) presents the associations between PGRS and WMM change. Panel (b) presents the regional associations between PGRS and cross-sectional regional WMM at time point one. Panel (c) presents the cross-sectional regional WMM associations with PGRS at time point two. Panel (d) presents cross-sectional regional associations between WMM and PGRS for the validation sample. Boxes the statistical significance at an uncorrected $\alpha < 0.05$. colors indicate the association strength (standardized β-coefficients). *Note:* All associations were adjusted for age, sex, the $age \times sex$ interaction, and site. None of the presented associations survived the adjustment of the α-level for multiple comparisons.

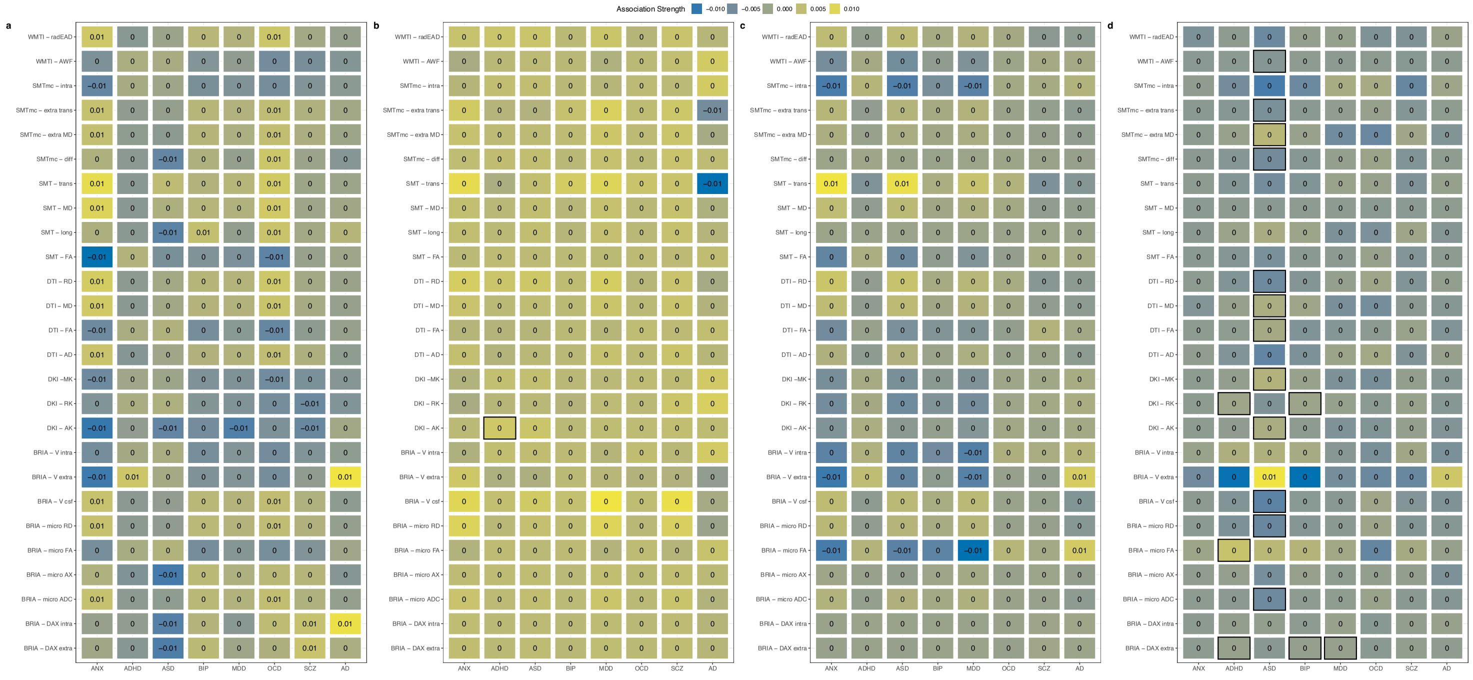

Appendix X Distribution of the relationship between PGRS and both the annual rate of WMM change as well as cross-sectional WMM.

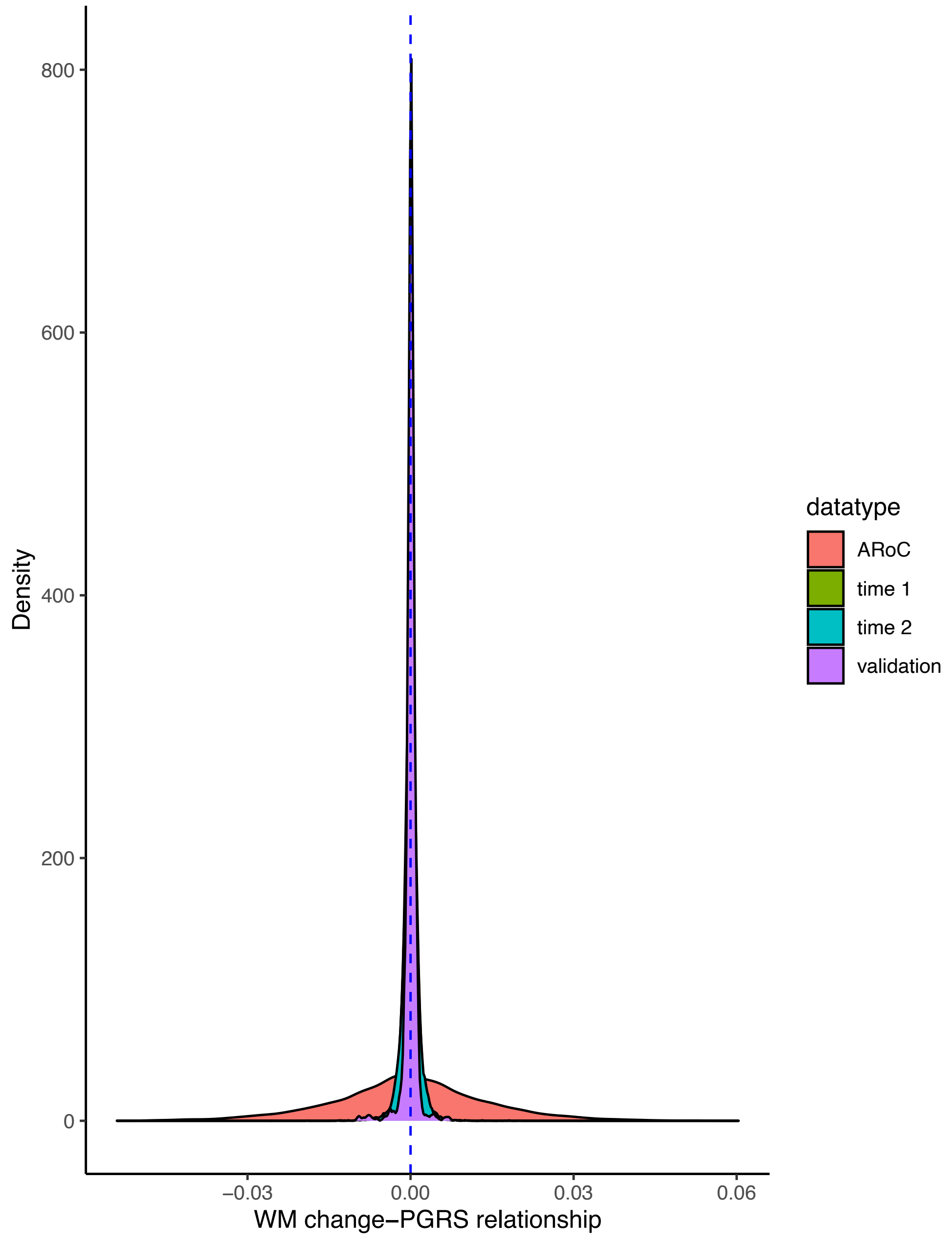

Appendix Y Voxel-level changes.

Voxel-level changes in BRIA - DAX extra.

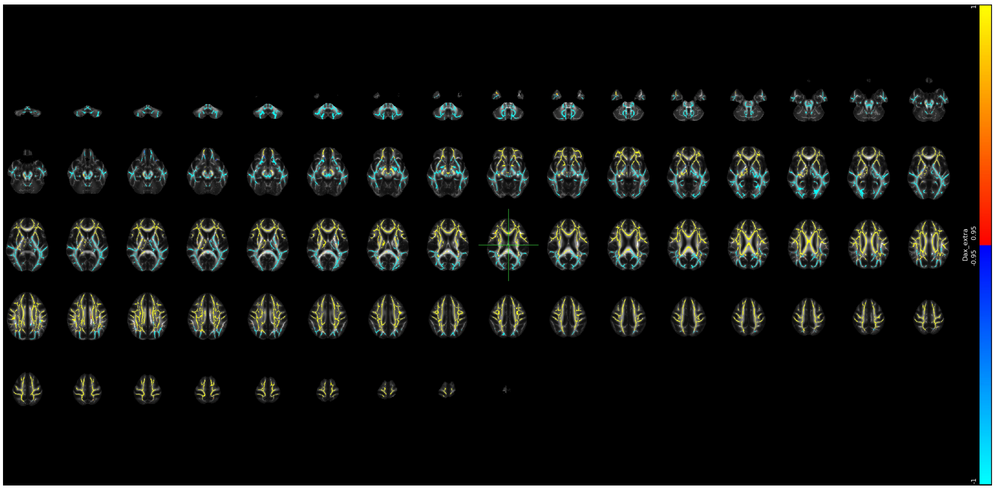

Voxel-level changes in BRIA - DAX intra

Red to yellow color indicate increases, dark to light blue colors decreases, where the scale is determined by the α-level ranging from 0.05 towards 0. MNI152 X:0, Y:-18, Z: 18 are indicated by the cursor with slices spacing of $\pm$ 4 steps along the z-axis in the coordinate system.

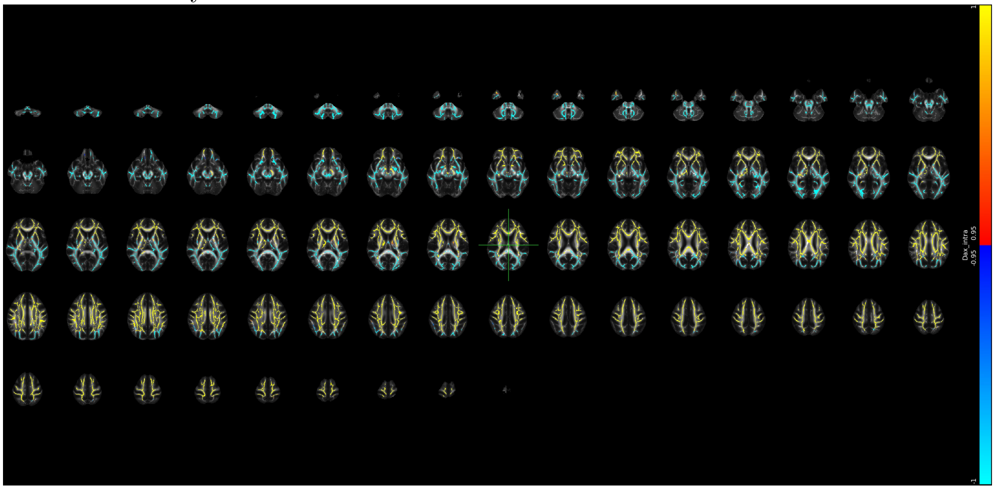

Voxel-level changes in BRIA - DAX intra.

Red to yellow color indicate increases, dark to light blue colors decreases, where the scale is determined by the α-level ranging from 0.05 towards 0. MNI152 X:0, Y:-18, Z: 18 are indicated by the cursor with slices spacing of $\pm$ 4 steps along the z-axis in the coordinate system.

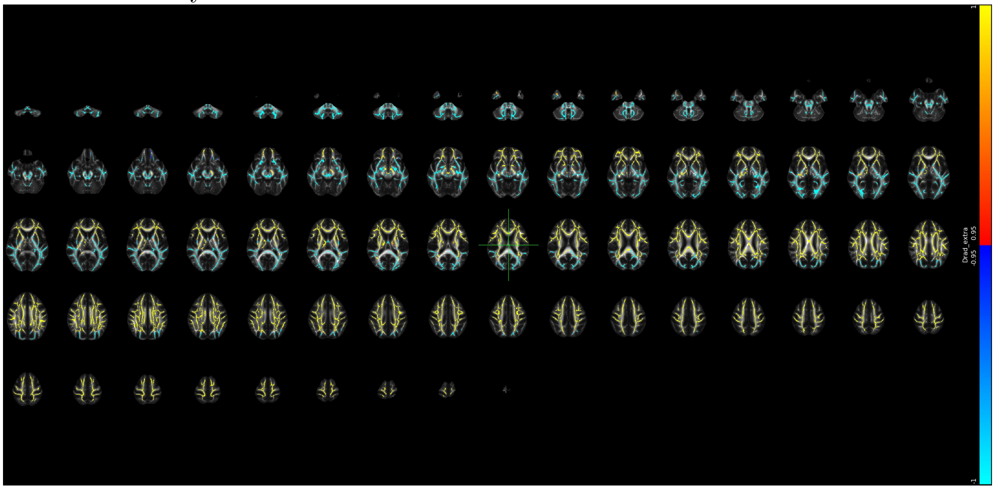

Voxel-level changes in BRIA - microADC.

Red to yellow color indicate increases, dark to light blue colors decreases, where the scale is determined by the α-level ranging from 0.05 towards 0. MNI152 X:0, Y:-18, Z: 18 are indicated by the cursor with slices spacing of $\pm$ 4 steps along the z-axis in the coordinate system.

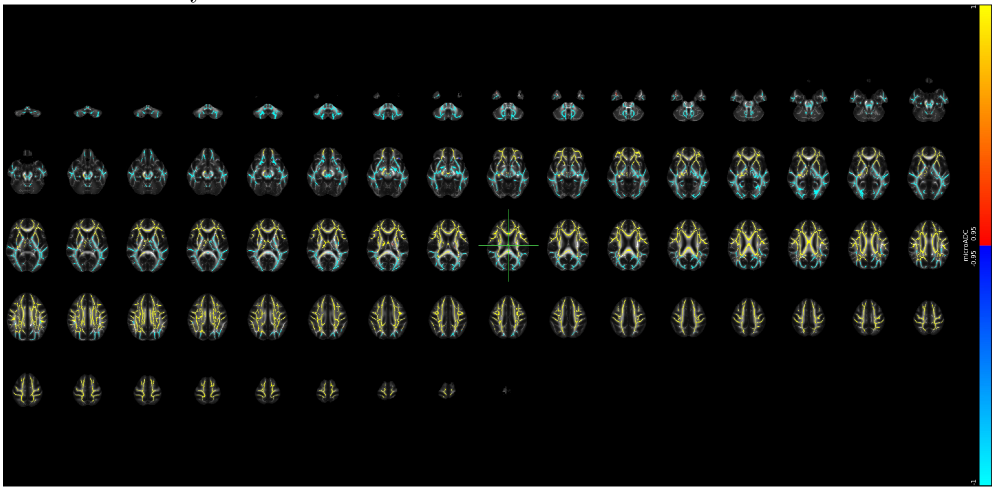

Voxel-level changes in BRIA - microAX.

Red to yellow color indicate increases, dark to light blue colors decreases, where the scale is determined by the α-level ranging from 0.05 towards 0. MNI152 X:0, Y:-18, Z: 18 are indicated by the cursor with slices spacing of $\pm$ 4 steps along the z-axis in the coordinate system.

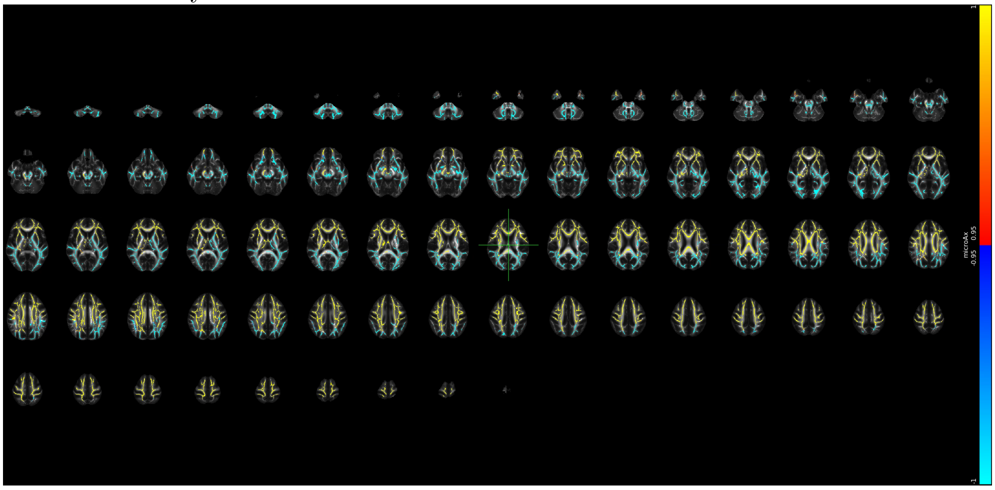

Voxel-level changes in BRIA - microFA.

Red to yellow color indicate increases, dark to light blue colors decreases, where the scale is determined by the α-level ranging from 0.05 towards 0. MNI152 X:0, Y:-18, Z: 18 are indicated by the cursor with slices spacing of $\pm$ 4 steps along the z-axis in the coordinate system.

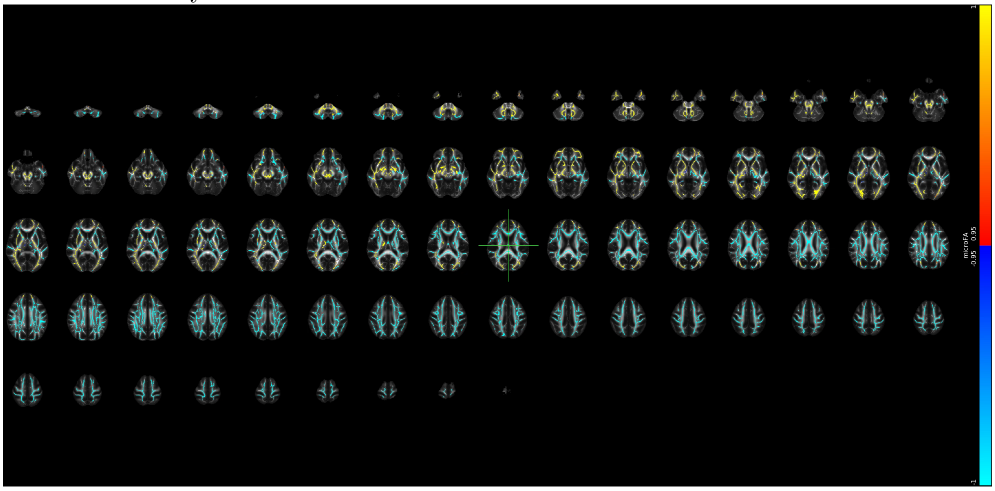

Voxel-level changes in BRIA - microRD.

Red to yellow color indicate increases, dark to light blue colors decreases, where the scale is determined by the α-level ranging from 0.05 towards 0. MNI152 X:0, Y:-18, Z: 18 are indicated by the cursor with slices spacing of $\pm$ 4 steps along the z-axis in the coordinate system.

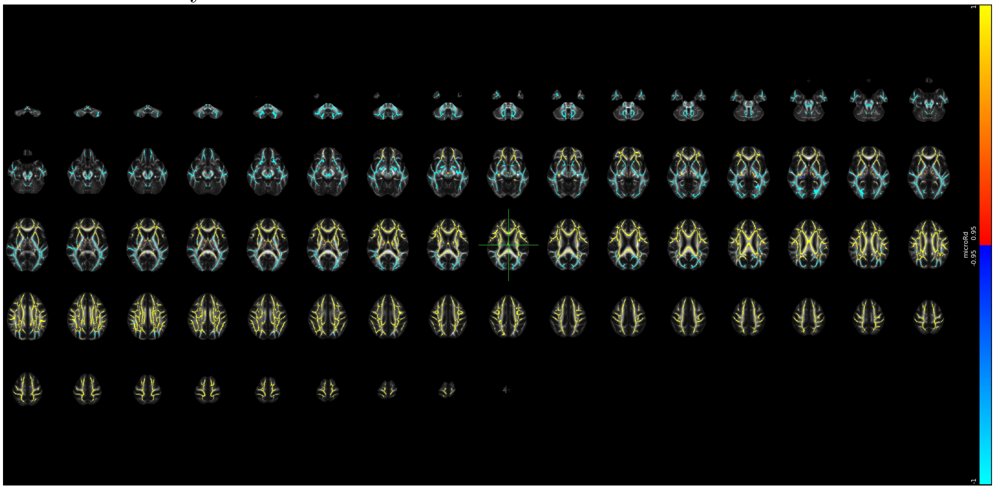

Voxel-level changes in BRIA - Vcsf.

Red to yellow color indicate increases, dark to light blue colors decreases, where the scale is determined by the α-level ranging from 0.05 towards 0. MNI152 X:0, Y:-18, Z: 18 are indicated by the cursor with slices spacing of $\pm$ 4 steps along the z-axis in the coordinate system.

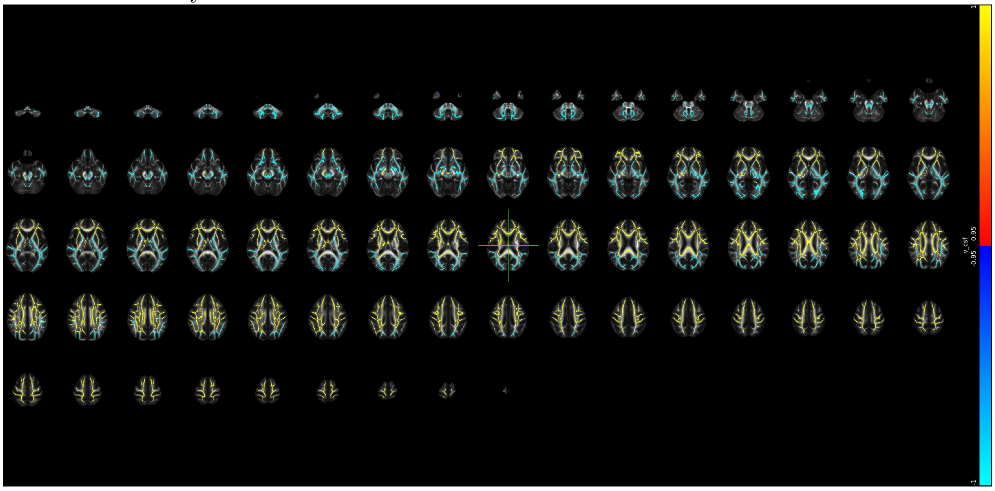

Voxel-level changes in BRIA - Vextra.

Red to yellow color indicate increases, dark to light blue colors decreases, where the scale is determined by the α-level ranging from 0.05 towards 0. MNI152 X:0, Y:-18, Z: 18 are indicated by the cursor with slices spacing of $\pm$ 4 steps along the z-axis in the coordinate system.

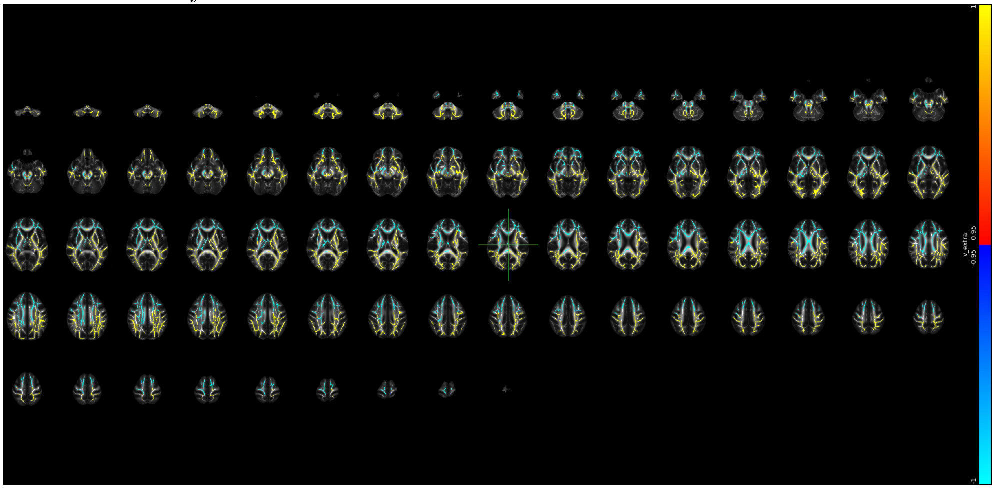

Voxel-level changes in BRIA - Vintra.

Red to yellow color indicate increases, dark to light blue colors decreases, where the scale is determined by the α-level ranging from 0.05 towards 0. MNI152 X:0, Y:-18, Z: 18 are indicated by the cursor with slices spacing of $\pm$ 4 steps along the z-axis in the coordinate system.

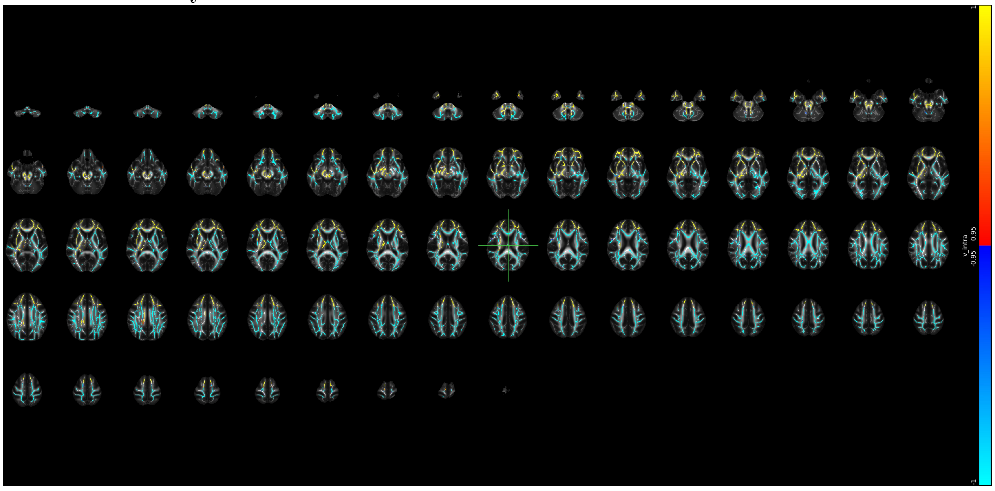

Voxel-level changes in DKI - AK.

Red to yellow color indicate increases, dark to light blue colors decreases, where the scale is determined by the α-level ranging from 0.05 towards 0. MNI152 X:0, Y:-18, Z: 18 are indicated by the cursor with slices spacing of $\pm$ 4 steps along the z-axis in the coordinate system.

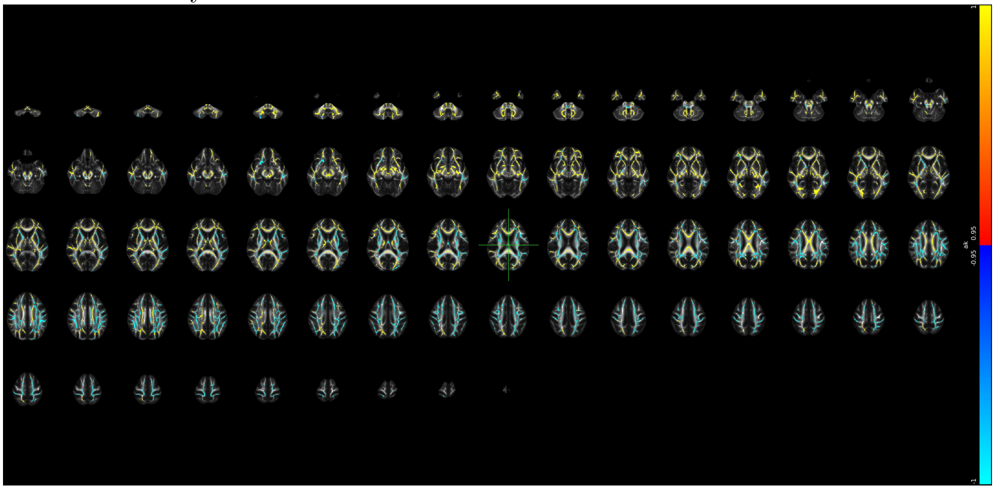

Voxel-level changes in DKI - MK.

Red to yellow color indicate increases, dark to light blue colors decreases, where the scale is determined by the α-level ranging from 0.05 towards 0. MNI152 X:0, Y:-18, Z: 18 are indicated by the cursor with slices spacing of $\pm$ 4 steps along the z-axis in the coordinate system.

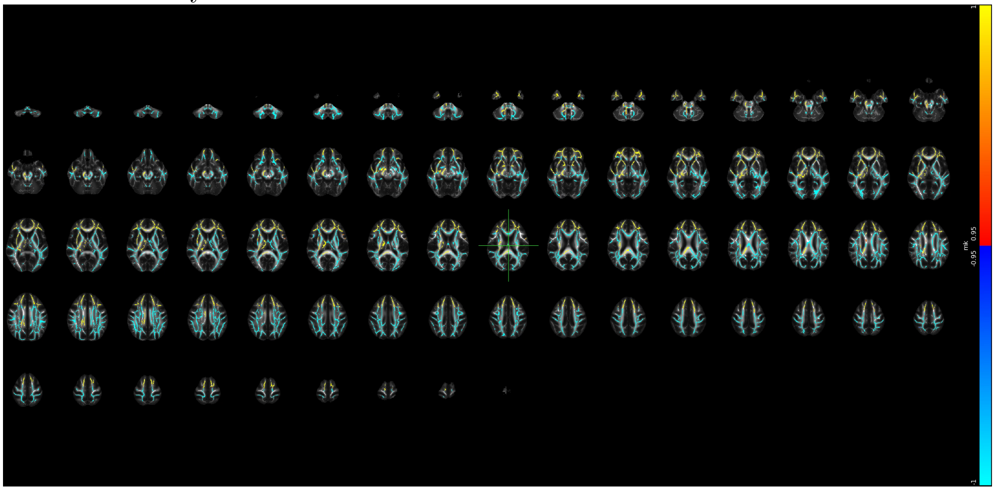

Voxel-level changes in DKI - RK.

Red to yellow color indicate increases, dark to light blue colors decreases, where the scale is determined by the α-level ranging from 0.05 towards 0. MNI152 X:0, Y:-18, Z: 18 are indicated by the cursor with slices spacing of $\pm$ 4 steps along the z-axis in the coordinate system.

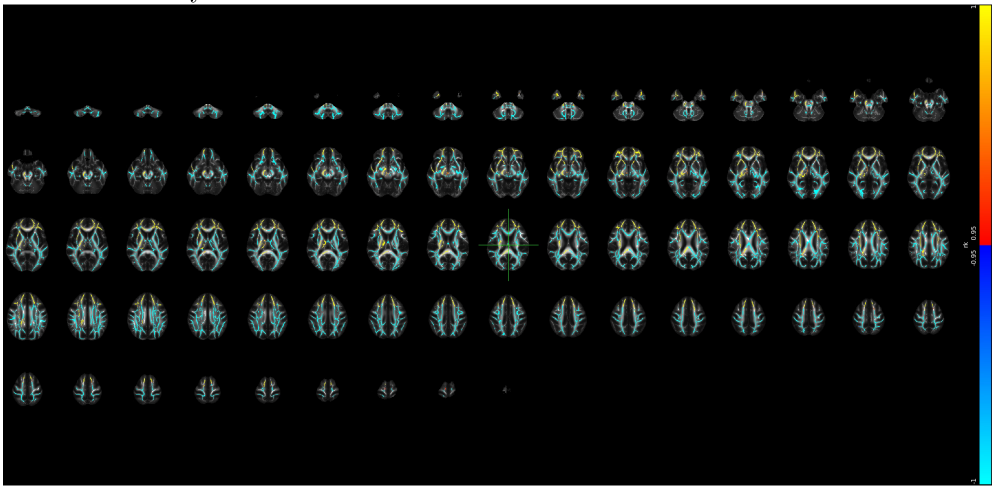

Voxel-level changes in DTI - AD.

Red to yellow color indicate increases, dark to light blue colors decreases, where the scale is determined by the α-level ranging from 0.05 towards 0. MNI152 X:0, Y:-18, Z: 18 are indicated by the cursor with slices spacing of $\pm$ 4 steps along the z-axis in the coordinate system.

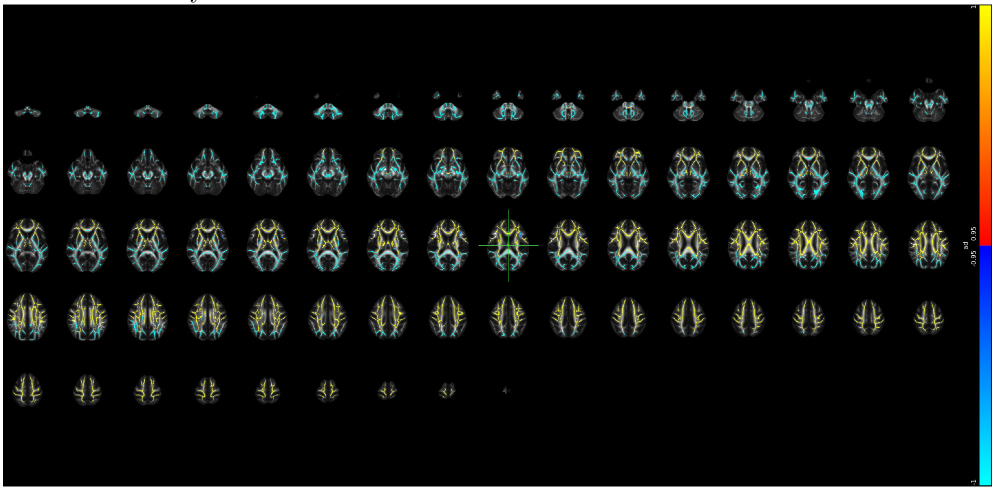

Voxel-level changes in DTI - FA.

Red to yellow color indicate increases, dark to light blue colors decreases, where the scale is determined by the α-level ranging from 0.05 towards 0. MNI152 X:0, Y:-18, Z: 18 are indicated by the cursor with slices spacing of $\pm$ 4 steps along the z-axis in the coordinate system.

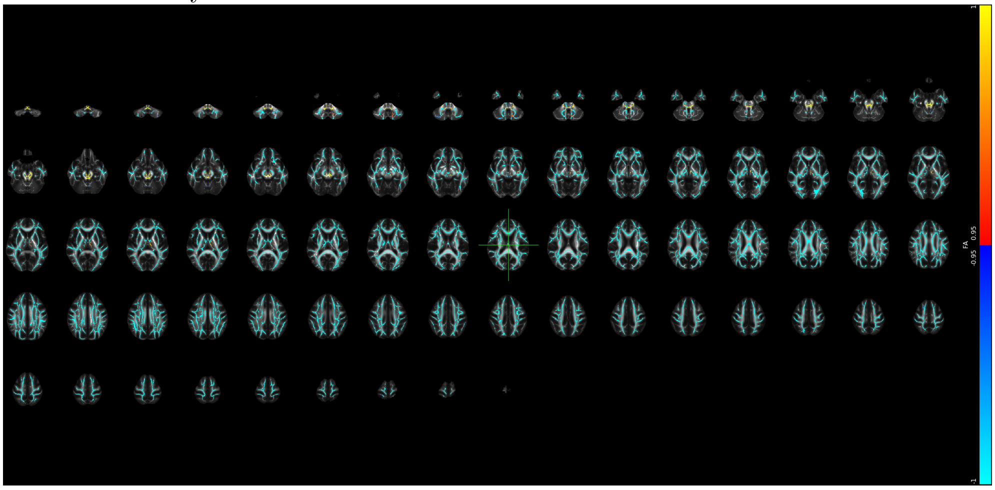

Voxel-level changes in DTI - MD.

Red to yellow color indicate increases, dark to light blue colors decreases, where the scale is determined by the α-level ranging from 0.05 towards 0. MNI152 X:0, Y:-18, Z: 18 are indicated by the cursor with slices spacing of $\pm$ 4 steps along the z-axis in the coordinate system.

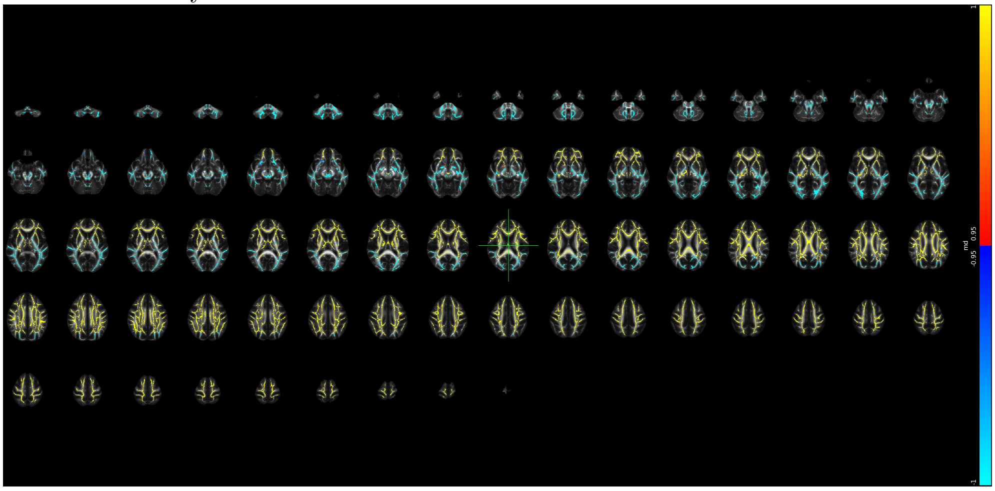

Voxel-level changes in DTI - RD.

Red to yellow color indicate increases, dark to light blue colors decreases, where the scale is determined by the α-level ranging from 0.05 towards 0. MNI152 X:0, Y:-18, Z: 18 are indicated by the cursor with slices spacing of $\pm$ 4 steps along the z-axis in the coordinate system.

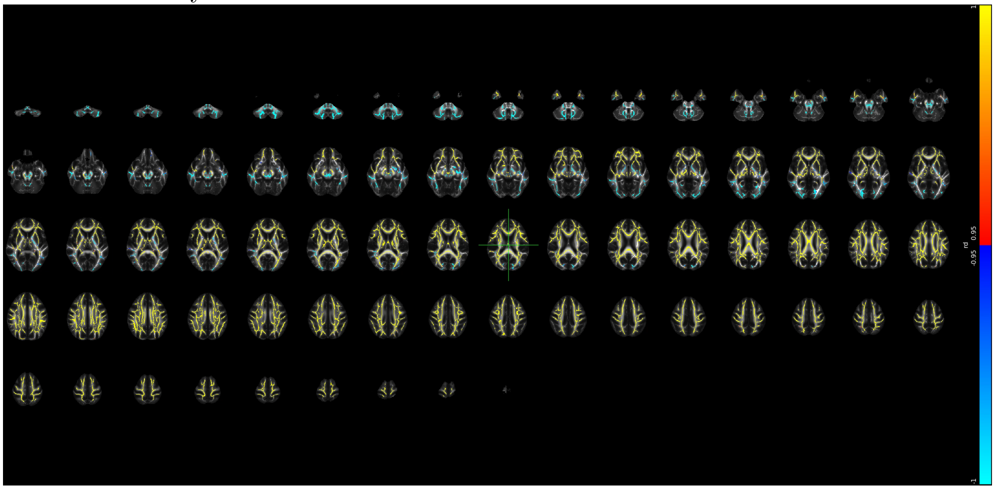

Voxel-level changes in SMTmc - diffusion coefficient.

Red to yellow color indicate increases, dark to light blue colors decreases, where the scale is determined by the α-level ranging from 0.05 towards 0. MNI152 X:0, Y:-18, Z: 18 are indicated by the cursor with slices spacing of $\pm$ 4 steps along the z-axis in the coordinate system.

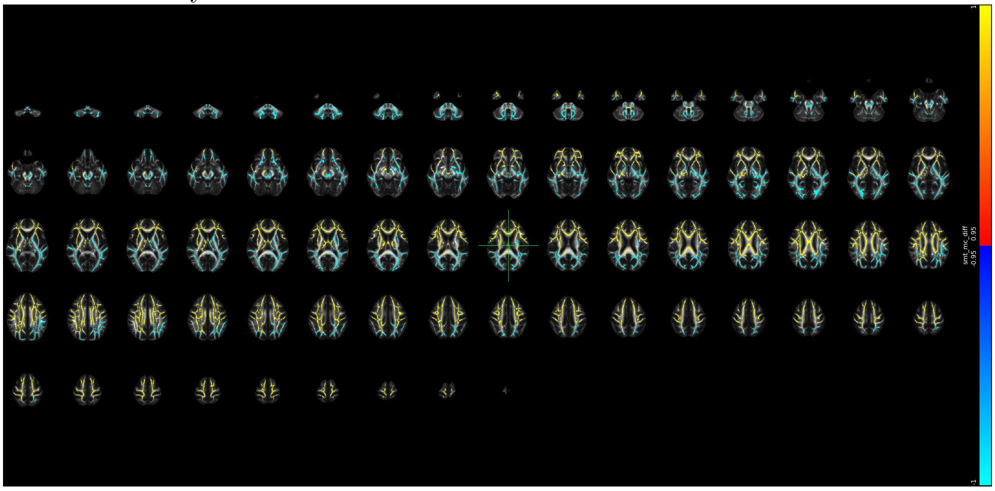

Voxel-level changes in SMTmc - extra MD.

Red to yellow color indicate increases, dark to light blue colors decreases, where the scale is determined by the α-level ranging from 0.05 towards 0. MNI152 X:0, Y:-18, Z: 18 are indicated by the cursor with slices spacing of $\pm$ 4 steps along the z-axis in the coordinate system.

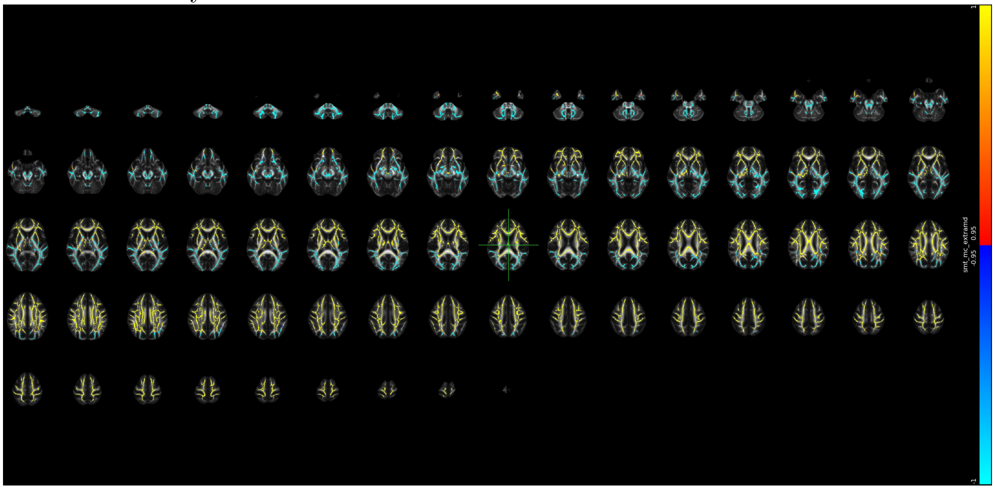

Voxel-level changes in SMTmc - extra trans.

Red to yellow color indicate increases, dark to light blue colors decreases, where the scale is determined by the α-level ranging from 0.05 towards 0. MNI152 X:0, Y:-18, Z: 18 are indicated by the cursor with slices spacing of $\pm$ 4 steps along the z-axis in the coordinate system.

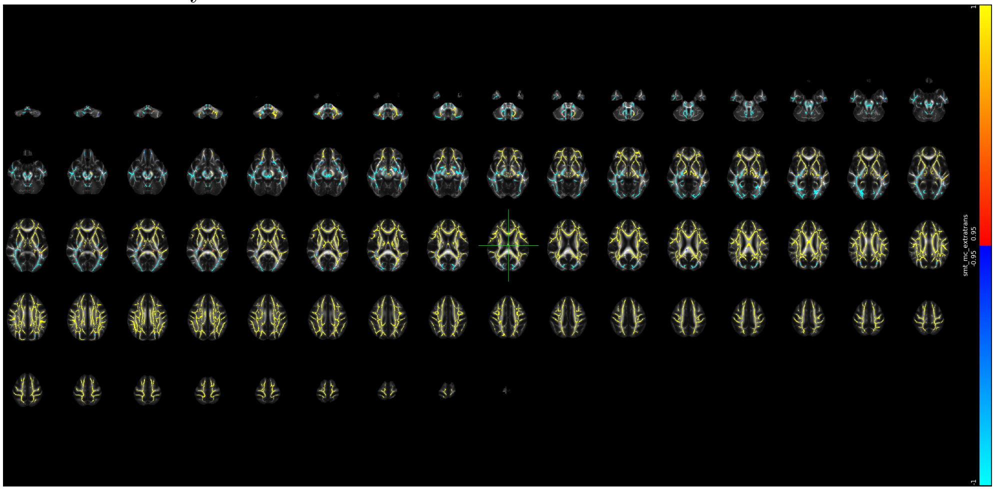

Voxel-level changes in SMTmc - intra.

Red to yellow color indicate increases, dark to light blue colors decreases, where the scale is determined by the α-level ranging from 0.05 towards 0. MNI152 X:0, Y:-18, Z: 18 are indicated by the cursor with slices spacing of $\pm$ 4 steps along the z-axis in the coordinate system.

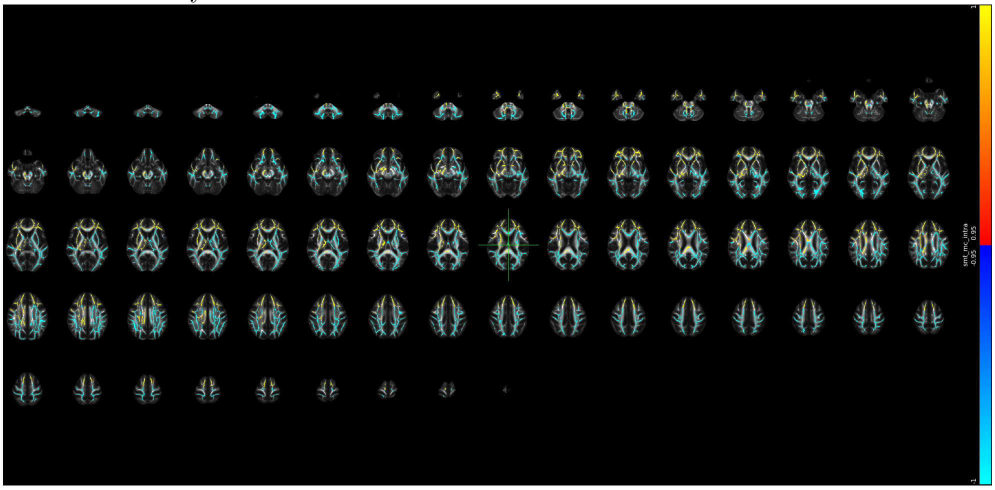

Voxel-level changes in WMT - AWF.

Red to yellow color indicate increases, dark to light blue colors decreases, where the scale is determined by the α-level ranging from 0.05 towards 0. MNI152 X:0, Y:-18, Z: 18 are indicated by the cursor with slices spacing of $\pm$ 4 steps along the z-axis in the coordinate system.

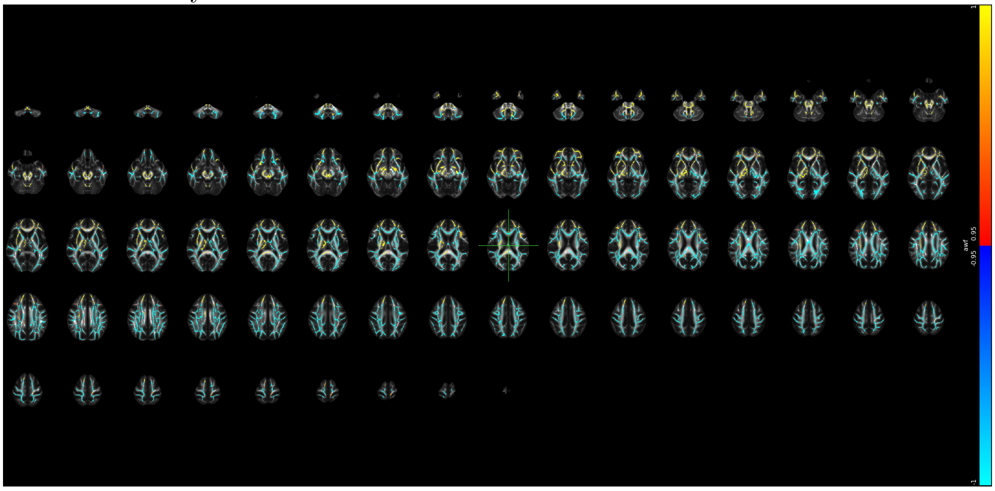

Voxel-level changes in WMTI - axEAD.

Red to yellow color indicate increases, dark to light blue colors decreases, where the scale is determined by the α-level ranging from 0.05 towards 0. MNI152 X:0, Y:-18, Z: 18 are indicated by the cursor with slices spacing of $\pm$ 4 steps along the z-axis in the coordinate system.

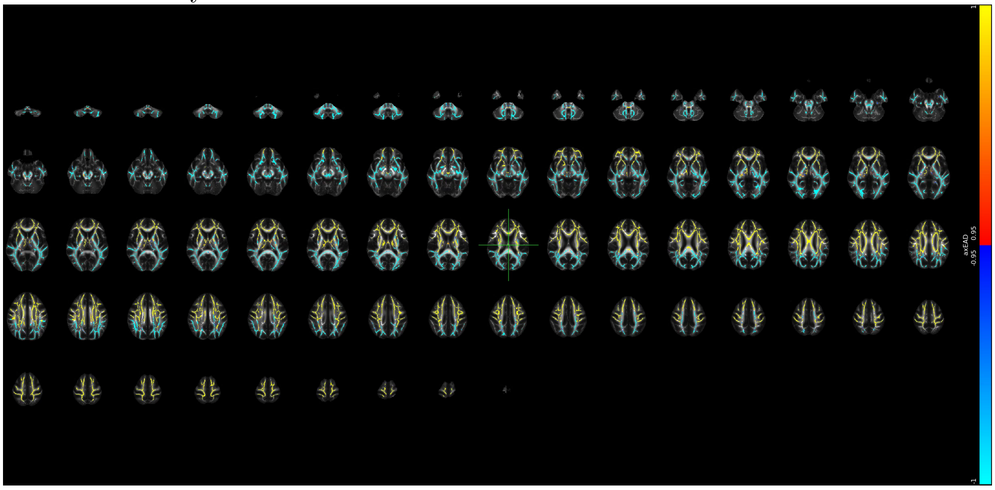

Voxel-level changes in WMTI - radEAD.

Red to yellow color indicate increases, dark to light blue colors decreases, where the scale is determined by the α-level ranging from 0.05 towards 0. MNI152 X:0, Y:-18, Z: 18 are indicated by the cursor with slices spacing of $\pm$ 4 steps along the z-axis in the coordinate system.

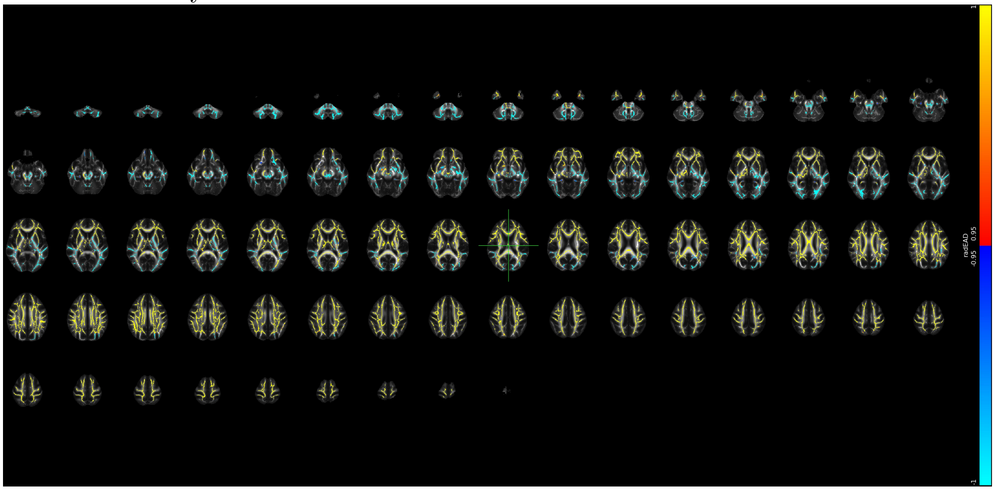

Voxel-level changes in SMT - FA.

Red to yellow color indicate increases, dark to light blue colors decreases, where the scale is determined by the α-level ranging from 0.05 towards 0. MNI152 X:0, Y:-18, Z: 18 are indicated by the cursor with slices spacing of $\pm$ 4 steps along the z-axis in the coordinate system.

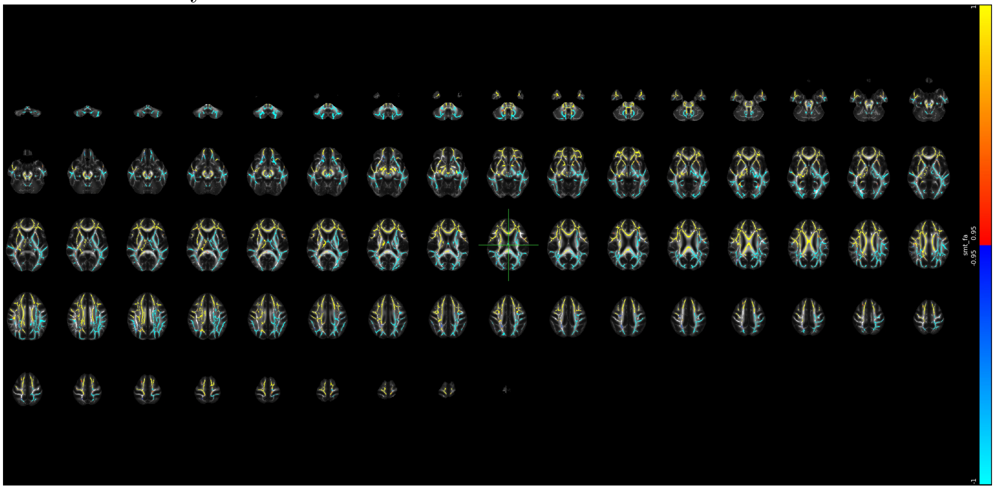

Voxel-level changes in SMT - MD.

Red to yellow color indicate increases, dark to light blue colors decreases, where the scale is determined by the α-level ranging from 0.05 towards 0. MNI152 X:0, Y:-18, Z: 18 are indicated by the cursor with slices spacing of $\pm$ 4 steps along the z-axis in the coordinate system.

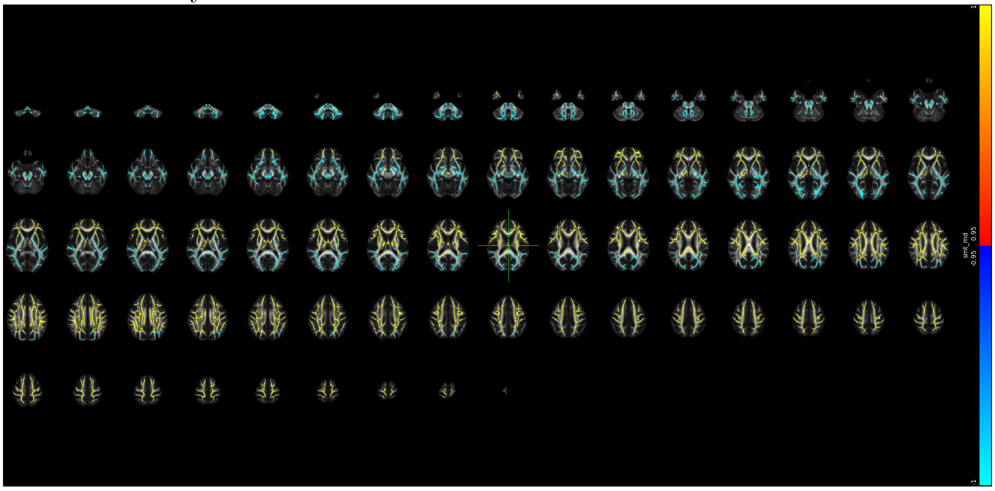

Voxel-level changes in SMT - longitudinal diffusion coefficient.

Red to yellow color indicate increases, dark to light blue colors decreases, where the scale is determined by the α-level ranging from 0.05 towards 0. MNI152 X:0, Y:-18, Z: 18 are indicated by the cursor with slices spacing of $\pm$ 4 steps along the z-axis in the coordinate system.

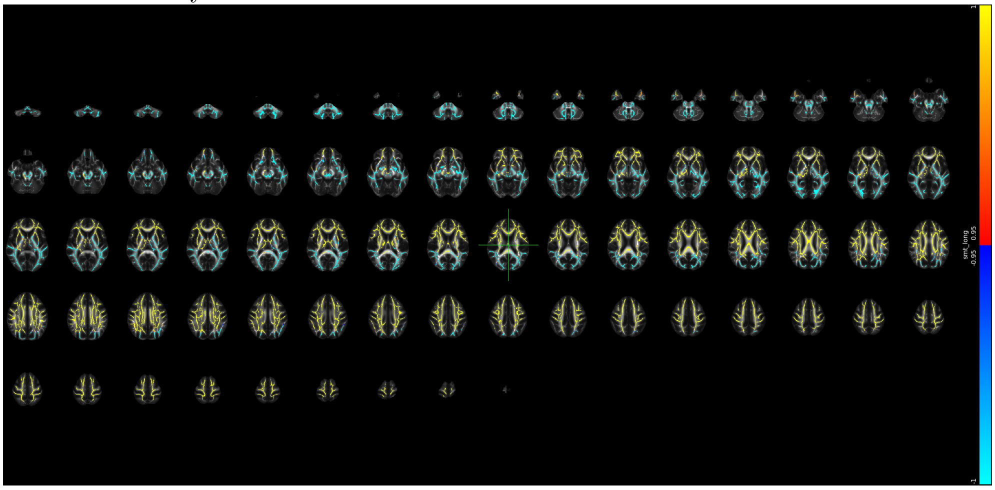

Voxel-level changes in SMT - transverse diffusion coefficient.

Red to yellow color indicate increases, dark to light blue colors decreases, where the scale is determined by the α-level ranging from 0.05 towards 0. MNI152 X:0, Y:-18, Z: 18 are indicated by the cursor with slices spacing of $\pm$ 4 steps along the z-axis in the coordinate system.

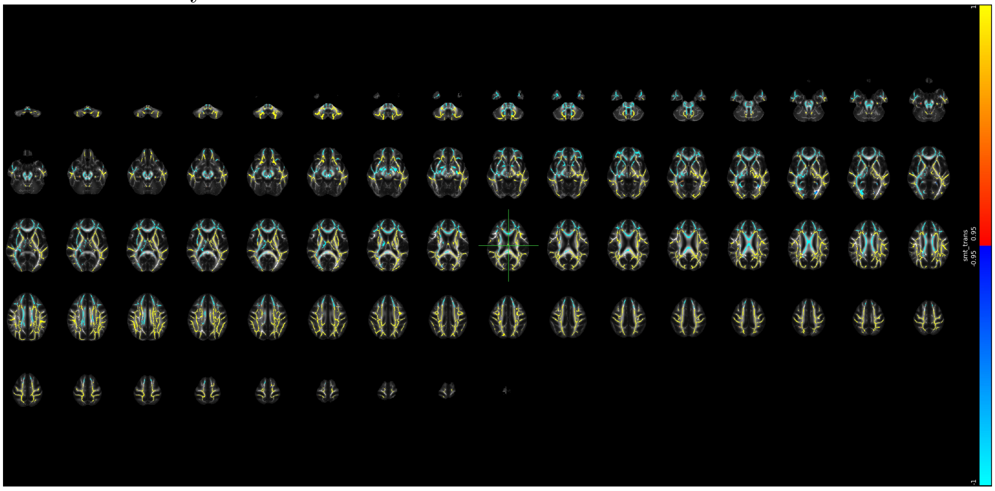

References

[1] Maximov et al. (2021), Fast qualitY conTrol meThod foR derIved diffUsion Metrics (YTTRIUM) in big data analysis: UK Biobank 18,608 example in Human Brain Mapping, 42(10).

[2] Wang et al. (2004), Image quality assessment: from error visibility to structural similarity in: IEEE 13(4).

[3] Brunet et al. (2011), On the mathematical properties of the structural similarity index in: IEEE 21(4).

[4] Reisert et al. (2017), Disentangling micro from mesostructure by diffusion MRI: a Bayesian approach in: NeuroImage, 147.

[5] Jensen et al. (2017), Diffusional kurtosis imaging: the quantification of non-gaussian water diffusion by means of magnetic resonance imaging in: MR in Med., 53(6).

[6] Fiermans et al. (2011), White matter characterization with diffusional kurtosis imaging in: NeuroImage, 58(1).

[7] Basser et al. (1994), MR diffusion tensor spectroscopy and imaging in: Biophys J., 66(1).

[8] Kaden et al. (2016), Quantitative mapping of the per-axon diffusion coefficients in brain white matter in: MR in Med., 75(4).

[9] Fiermans et al. (2011), White matter characterization with diffusional kurtosis imaging in: NeuroImage, 58(1).

[10] Kaden et al. (2016), Multi-compartment microscopic diffusion imaging in: NeuroImage, 139.
